# H2AK121ub in *Arabidopsis* associates with a less accessible chromatin state at transcriptional regulation hotspots

**DOI:** 10.1038/s41467-020-20614-1

**Published:** 2021-01-12

**Authors:** Xiaochang Yin, Francisco J. Romero-Campero, Pedro de Los Reyes, Peng Yan, Jing Yang, Guangmei Tian, XiaoZeng Yang, Xiaorong Mo, Shuangshuang Zhao, Myriam Calonje, Yue Zhou

**Affiliations:** 1 State Key Laboratory of Protein and Plant Gene Research, School of Advanced Agricultural Sciences, Peking-Tsinghua Center for Life Sciences, Peking University, 100871 Beijing, China; 2 Institute of Plant Biochemistry and Photosynthesis (IBVF-CSIC), Avenida Américo Vespucio 49, 41092 Seville, Spain; 3 Department of Computer Science and Artificial Intelligence (University of Sevilla), Avenida Reina Mercedes s/n, 41012 Seville, Spain; 4 State Key Laboratory of Plant Physiology and Biochemistry, College of Life Science, Zhejiang University, 310058 Hangzhou, China; 5 Beijing Agro-biotechnology Research Center, Beijing Key Laboratory of Agricultural Genetic Resources and Biotechnology, Beijing Academy of Agriculture and Forestry Sciences, 100097 Beijing, China; 6 Academy for Advanced Interdisciplinary Studies, Peking University, 100871 Beijing, China; 7 Key Laboratory of Plant Stress, Life Science College, Shandong Normal University, 250014 Jinan, China

**Keywords:** DNA methylation, Cell fate, Plant molecular biology

## Abstract

Although it is well established that the Polycomb Group (PcG) complexes maintain gene repression through the incorporation of H2AK121ub and H3K27me3, little is known about the effect of these modifications on chromatin accessibility, which is fundamental to understand PcG function. Here, by integrating chromatin accessibility, histone marks and expression analyses in different *Arabidopsis* PcG mutants, we show that PcG function regulates chromatin accessibility. We find that H2AK121ub is associated with a less accessible but still permissive chromatin at transcriptional regulation hotspots. Accessibility is further reduced by EMF1 acting in collaboration with PRC2 activity. Consequently, H2AK121ub/H3K27me3 marks are linked to inaccessible although responsive chromatin. In contrast, only-H3K27me3-marked chromatin is less responsive, indicating that H2AK121ub-marked hotspots are required for transcriptional responses. Nevertheless, despite the loss of PcG activities leads to increased chromatin accessibility, this is not necessarily accompanied by transcriptional activation, indicating that accessible chromatin is not always predictive of gene expression.

## Abstract (web summary)

Polycomb Group complexes maintain gene repression through the incorporation of H2AK121ub and H3K27me3. Here, the authors show that H2AK121ub marks less accessible but transcriptionally permissive chromatin, while H3K27me3 enforces a repressed transcriptionally less-permissive state.

## Introduction

PcG complexes maintain gene repression by incorporating histone modifications within chromatin^1–3^. While PcG repressive complex 1 (PRC1) has histone 2A E3 ubiquitin ligase activity^4–8^, PRC2 has histone 3 lysine 27 (H3K27) trimethyltransferase activity^9–12^. Nevertheless, despite the important role of these chromatin marks in regulating transcription in eukaryotes, their effect on chromatin is not yet clear. Several pieces of evidence suggested that animal PcG proteins mediate chromatin compaction^13–15^. However, a recent report proposed that although PcG occupied promoters exhibit reduced accessibility, this does not rely on PcG proteins. Instead, PRC1 plays a role in increasing nucleosome occupancy and decreasing nucleosomal spacing^16^.

In *Arabidopsis thaliana* (*Arabidopsis*), the composition of PcG complexes is still a matter of debate. While PRC2 core components are well conserved to their animal counterparts and the catalytic activity is mainly carried out by the EZ homologs CURLY LEAF (CLF) and SWINGER (SWN) during sporophyte development^3,17^, the identity of PRC1 is less clear. Despite the H2A monoubiquitination module is formed by the Polycomb Group ring finger (PCGF) and RING1 homologs BMI1 (A, B, or C) and RING1 (A or B)^2,18^, association of the plant-specific proteins EMBRYONIC FLOWER 1 (EMF1) and LIKE-HETEROCHROMATIN PROTEIN 1 (LHP1) to this complex has turned to be controversial. EMF1 was proposed to be a PRC1 component due to its ability to in vitro mediate chromatin compaction as *Drosophila melanogaster (Drosophila)* PRC1 component Posterior Sex Combs (Psc) does^19,20^. However, recent data showed that EMF1 co-purifies with PRC2 (refs. ^21–23^) and is required for H3K27me3 marking^19,24,25^. Likewise, LHP1 was proposed to perform similar function to *Drosophila* PRC1 component Polycomb (Pc)^26,27^; however, LHP1 also co-purifies with PRC2 (refs. ^21–23^) and is involved in maintenance/spreading of H3K27me3 (ref. ^21^) but dispensable for H2AK121ub marking^28^. Thus, these two proteins actually seem to be PRC2-associated component.

Interestingly, although H2AK121ub and H3K27me3 marks often co-localize at genes (H2AK121ub/H3K27me3-marked genes), H3K27me3-marked peaks are generally much longer than that of H2AK121ub^28^. Accordingly, H3K27me3 marks usually cover the complete gene region, whereas H2AK121ub marks are enriched at the region surrounding the transcriptional start site (TSS)^28^. In addition, there are also genes marked with only-H2AK121ub or H3K27me3 (ref. ^28^) (only-H2AK121ub and only-H3K27me3, respectively), indicating that these marks may play independent roles.

H3K27me3 marks in *Arabidopsis* have been linked to regions displaying locally reduced DNA accessibility^29^. According to this, the promoters of expressed genes display high chromatin accessibility and are depleted of H3K27me3 marks^30,31^. However, it is not clear whether H3K27me3 has a direct role in regulating chromatin accessibility. On the other hand, the role of PRC1-mediated H2AK121ub is far from clear. Although several data support a repressive role of this modification^6,8,28^, it has been proposed to be associated with gene responsiveness and its repressive function seems to require PRC2 recruitment^32^. In any case, nothing is known about a possible role of H2AK121ub in regulating chromatin accessibility in plants.

In this work, by integrating chromatin accessibility, histone marks, and expression analyses in different *Arabidopsis* PcG mutants, we show that H2AK121ub marks associate with a less accessible but permissive chromatin at transcriptional regulation hotspots, which are sites enriched for the binding of transcription factors (TFs). Chromatin accessibility can be further reduced by EMF1 and PRC2 activity. However, while H2AK121ub/H3K27me3-mediated inaccessible chromatin is still transcriptionally responsive, only-H3K27me3 marked chromatin is less responsive.

## Results

### H2AK121ub hallmarks hotspots for transcriptional regulation

To investigate a possible role of H3K27me3 and H2AK121ub in regulating chromatin accessibility, we first performed Assay for Transposase-Accessible Chromatin sequencing (ATAC-seq) in wild-type Col-0 (WT) and different PcG mutants at 10 days after germination (DAG). We selected *bmi1abc*, *clf28swn7,* and *emf1-2* severe mutants^8,19,33^ that cannot undergo the transition from embryonic to vegetative development after germination in the aerial part of the seedling. However, unlike *bmi1abc* and *clf28swn7*, which in addition have a stunted root displaying embryonic traits, *emf1-2* is able to develop a WT-like root (Supplementary Fig. 1). We in addition included *ring1ab* weak and *lhp1* mutants that are able to develop vegetative and floral organs in spite of several developmental alterations^34,35^ (Supplementary Fig. 1).

**Fig. 1:**
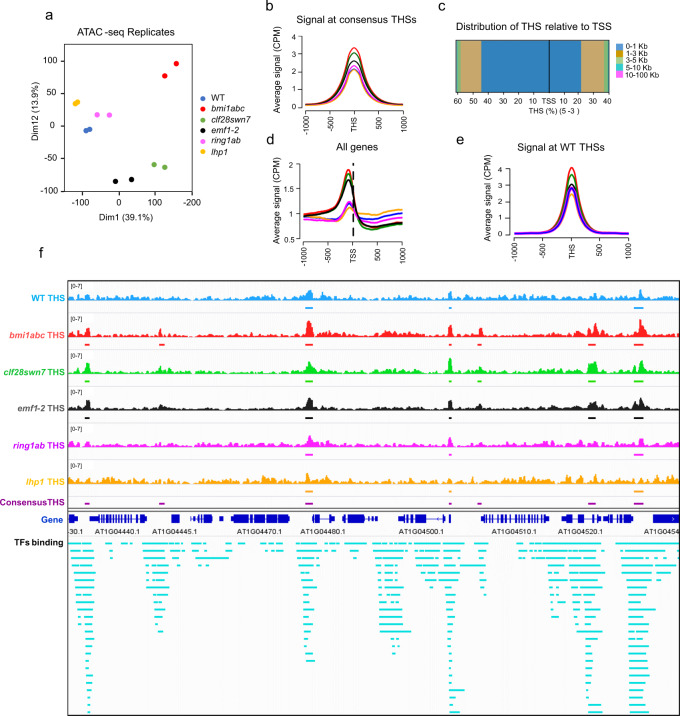
Consensus THSs co-localize with transcriptional regulation hotspots. **a** PCA analysis of WT, *bmi1abc*, *clf28swn7*, *emf1-2*, *lhp1*, and *ring1ab* ATAC-seq replicates at 10 DAG. **b** Average signal levels at the center of consensus THSs in WT, *bmi1abc*, *clf28swn7*, *emf1-2*, *lhp1*, and *ring1ab* mutants. **c** Distribution of consensus THSs relative to TSS. The percentage of THSs found in different intervals is indicated in different colors. **d** Average accessibility profile of coding genes at the region surrounding the TSS in WT and mutants. **e** Average signal levels at the center of WT THSs in WT, *bmi1abc*, *clf28swn7*, *emf1-2*, *lhp1*, and *ring1ab* mutants. **f** Screenshot showing distribution of genotype-specific THSs, consensus THSs, and TFs binding sites (see also Supplementary Data 2). Each genotype is indicated in different color (WT in blue, *bmi1abc* in red, *clf28swn7* in green, *emf1-2* in black, *ring1ab* in pink, and *lhp1* in orange). Consensus THSs are indicated in magenta. Gene regions are indicated in dark blue and the binding sites of TFs in greenish blue.

Principal component analysis (PCA) of ATAC-seq data revealed that WT, *ring1ab*, and *lhp1* clustered together, whereas *clf28swn7* and *emf1-2* on one side, and *bmi1abc* on the other, constituted two distant and distinct clusters, indicating differences between them (Fig. 1a). We next identified Tn5 hypersensitive sites (THSs) for each genotype as those genomic regions exhibiting a significant accumulation of signal from the corresponding ATAC-seq data in the two different replicates. We found that the number of THSs in WT was lower than that in the different mutants (11,351 in WT, 12,803 in *lhp1*, 13,829 in *ring1ab*, 13,472 in *emf1-2*, 17,162 in *clf28swn7*, and 17,420 in *bmi1abc*; Supplementary Fig. 2). In addition, most of WT THSs were also accessible regions in mutants, indicating that loss-of-function of these PcG proteins leads to emergence of new THSs (Supplementary Fig. 2). To compare the contribution of the different mutants in increasing chromatin accessibility, THSs from the different genotypes were merged together into a list of consensus THSs. The list consisted in highly confident consensus THSs exhibiting an accessibility signal greater than 3 CPM with a *q*-value <0.05 in at least one of the genotypes (17,372; Supplementary Data 1). Average accessibility signal at consensus THSs was much higher in severe mutants than in *ring1ab* and *lhp1*, in which the signal was similar to WT (Fig. 1b). Consensus THSs were mostly localized within the 1 kb region upstream of the TSS (Fig. 1c). Accordingly, we found an accessibility signal peak at the region prior to the TSS in all genotypes (Fig. 1d). We then analyzed the average accessibility signal at WT THSs in the different genotypes (Fig. 1e). Interestingly, the embryonic mutants *bmi1abc*, *clf28swn7*, and *emf1-2* showed higher accessibility signals at this subset of THSs than the genotypes that develop vegetative tissues, as is the case of WT, *ring1ab*, and *lhp1* mutants. This is consistent with the fact that undifferentiated embryonic cells tend to have a more accessible chromatin state than differentiated cells^36–38^.

We found that consensus THSs co-localized with regions highly enriched in transcription factor (TF)-binding sites (enrichment = 7.5, *p* value <10^−4^; Fig. 1f) as determined by genome-wide identification of the binding sites of 100 TFs (Supplementary Data 2), supporting that THSs indicate the presence of transcriptional regulation hotspots^30,36,39^. According to this, a high number of transcriptional regulation hotspots that were inaccessible in WT became accessible in one or more mutants (Fig. 1f).

**Fig. 2:**
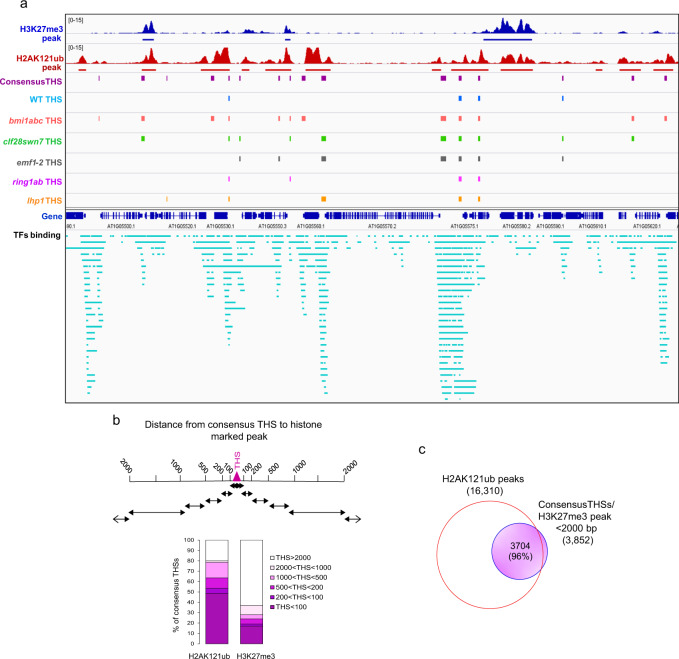
H2AK121 monoubiquitination hallmarks hotspots for transcriptional regulation. **a** Screenshots showing distribution of H3K27me3 and H2AK121ub peaks in WT, consensus THSs, genotype-specific THSs, and TFs binding sites. H3K27me3 and H2AK121ub peaks are indicated in blue and red, respectively. Consensus THSs, genotype-specific THSs, gene regions, and TFs binding sites color code is as in Fig. 1f. **b** Percentage of consensus THSs showing an H2AK121ub or H3K27me3 region at different non-overlapping distance intervals as indicated. **c** Venn diagram showing the overlap between consensus THSs having an H3K27me3 peak within the next 2 kb and H2AK121ub peaks.

Nevertheless, the emergence of new THSs in mutants could be the result of a direct or indirect consequence of loss of PcG regulation. Therefore, we compared the genome-wide distribution of H2AK121ub and H3K27me3 marks in WT to that of consensus THSs, genotype-specific THSs, and TFs binding sites (Fig. 2a). We found that consensus THSs tend to associate with PcG-marked regions (Fig. 2a and Supplementary Fig. 3). Intriguingly, around 80% of consensus THSs showed an H2AK121ub peak in its vicinity (≤2 kb distance from THS) (Fig. 2a, b); furthermore, 50% of consensus THSs overlapped or had an H2AK121ub peak at less than 100 bp away (Fig. 2b). In contrast, this was not so evident for H3K27me3, as only around 40% of consensus THSs showed an H3K27me3 peak within the next 2 kb (Fig. 2b) and 96% of these regions co-localized with H2AK121ub (Fig. 2c). All together, these results strongly suggest that the loss of PcG function plays a role in increasing accessibility at transcriptional regulation hotspots; moreover, that H2AK121 monoubiquitination hallmarks these hotspots.

### H2AK121ub and H3K27me3 are differentially affected in PcG mutants

**Fig. 3:**
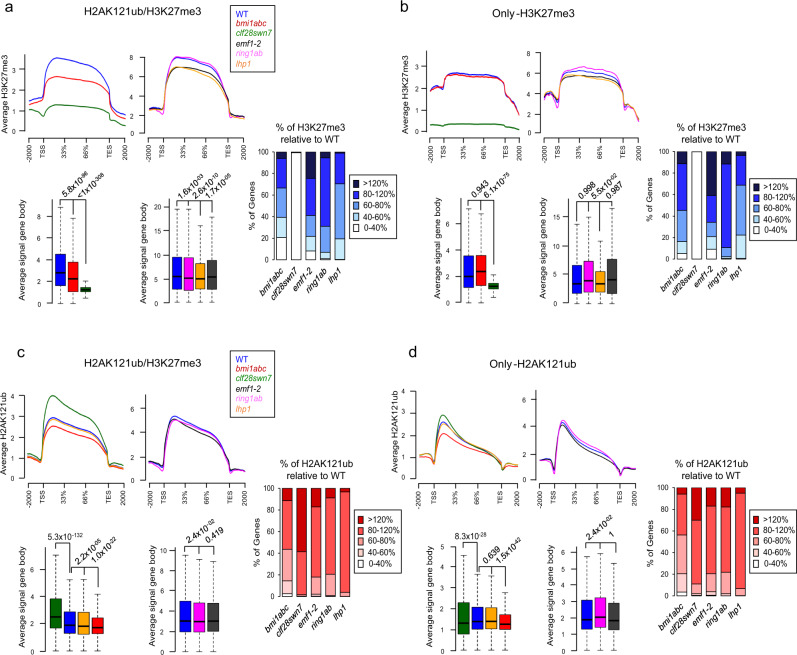
H2AK121ub and H3K27me3 levels in mutant and WT seedlings. **a** Metagene plots of H3K27me3 coverage at H2AK121ub/H3K27me3-marked genes in the different mutants. Left panel shows results using previously published data^28^ and right panel using data generated in this work (see “Methods”). Bar plots indicate the percentage of genes displaying different levels of H3K27me3 marks relative to WT in each mutant. Box plots below metagene plots show average signal at gene body in the different genotypes. The median (middle line), upper and lower quartiles (boxes), and minimum and maximum values (whiskers) are indicated. *P* values of differences between WT and mutants according to one-sided Mann–Whitney–Wilcoxon test are also indicated. **b** Same as in **a** but for only-H3K27me3-marked genes. Box plots below metagene plots show average signal at gene body in the different genotypes. The median (middle line), upper and lower quartiles (boxes), and minimum and maximum values (whiskers) are indicated. *P* values of differences between WT and mutants according to one-sided Mann–Whitney–Wilcoxon test are indicated. **c** Metagene plots of H2AK121ub coverage at H2AK121ub/H3K27me3-marked genes in the different mutants. Left panel results using previously published data^28^ and right panel using data generated in this work (see “Methods”). Bar plots indicate the percentage of genes displaying different levels of H2AK121ub marks relative to WT in each mutant. Box plots below metagene plots show average signal at gene body in the different genotypes. The median (middle line), upper and lower quartiles (boxes), and minimum and maximum values (whiskers) are indicated. *P* values of differences between WT and mutants according to one-sided Mann–Whitney–Wilcoxon test are indicated. **d** Same as in **c** but for only-H2AK121ub-marked genes. Box plots below metagene plots show average signal at gene body in the different genotypes. The median (middle line), upper and lower quartiles (boxes), and minimum and maximum values (whiskers) are indicated. *P* values of differences between WT and mutants according to one-sided Mann–Whitney–Wilcoxon test are indicated. In all cases (**a**–**d**), data were generated from *n* = 2 biological independent ChIP-seq samples. The list of only-H2AK121ub, H2AK121ub/H3K27me3, and only-H3K27me3 marked genes are provided as a Source Data file.

To investigate the role of H3K27me3 and H2AK121ub marks in regulating chromatin accessibility, we next analyzed the signal levels of these modifications at H2AK121ub/H3K27me3, only-H3K27me3, and only-H2AK121ub marked genes in the different mutants (Fig. 3a–d). For this, we used previously published ChIP-seq data for some of the mutants and WT^28^, and generated new datasets for the remaining mutants together with a new WT dataset (see “Methods” section). We redefined H2AK121ub/H3K27me3, only-H3K27me3, and only-H2AK121ub marked genes as those genes displaying the corresponding modification/s in one of the two WT datasets, resulting in similar gene lists than the ones previously published^28^ (see “Methods” section).

Average H3K27me3 levels at H2AK121ub/H3K27me3-marked genes were almost undetectable in *clf28swn7* and significantly decreased in *bmi1abc* compared to WT as previously described^28^ (Fig. 3a), which is consistent with a requirement of PRC1 activity for H3K27me3 marking. Accordingly, ChIP-reChIP experiments using anti-H3K27me3 and anti-H2AK121ub antibodies verified that these marks co-localize at some nucleosomes of H2AK121ub/H3K27me3-marked genes (Supplementary Fig. 4). We also found reduced levels of H3K27me3 in *lhp1* and *emf1-2* at these genes, although the differences with respect to WT were less significant than in *bmi1abc*. Conversely, *ring1ab* weak mutant showed WT-like levels (Fig. 3a). Nevertheless, analyzing the levels of H3K27me3 at individual genes in *ring1ab*, we found specific genes displaying less than 60% of WT levels (Fig. 3a and Supplementary Fig. 5), which is consistent with previous results^40^. The low percentage of genes showing strongly reduced levels of H3K27me3 in *ring1ab* (around 7%) is most probably due to the presence of RING1 activity in this knockdown mutant. All together, these data indicate that all these proteins are required for appropriate H3K27me3 marking at H2AK121ub/H3K27me3 genes. On the contrary, average global H3K27me3 levels at only-H3K27me3 genes were significantly affected in *clf28swn7* and *lhp1* but not in strong *bmi1abc*, weak *ring1ab*, and unexpectedly *emf1-2* (Fig. 3b).

Interestingly, despite we found decreased levels of H3K27me3 at many genes in *emf1-2*, which supports that EMF1 plays an important role in H3K27me3 deposition^19,24,25^, we also found a high percentage of both H2AK121ub/H3K27me3 and only-H3K27me3 genes with increased levels of H3K27me3 in *emf1-2* compared to WT (Fig. 3a, b and Supplementary Fig. 6). Phenotypic analysis of the different mutants indicated that unlike BMI1 and CLF/SWN, EMF1 might not be decisive for the incorporation of H3K27me3 marks in root tissue as *emf1-2* can develop a WT-like root (Supplementary Fig. 1). Since we used whole seedlings for ChIP-seq analyses, the fact that *emf1-2* displays a stunted shoot development but a WT-like root implies a higher proportion of root cells in *emf1-2* samples compared to WT, which develops shoot and root tissues. Therefore, the apparently increased levels of H3K27me3 at some genes in *emf1-2* might be a consequence of the different ratio of root cells between *emf1-2* and WT samples, which lead to increased average H3K27me3 levels in *emf1-2* at root repressed genes. Supporting this, Gene Ontology (GO) analysis of genes with increased H3K27me3 levels in *emf1-2* showed an enrichment in processes involved in photosynthesis and response to light, corresponding to genes marked with H3K27me3 in both WT and *emf1-2* roots (Supplementary Fig. 6).

Regarding H2AK121ub marks, average H2AK121ub signal levels at H2AK121ub/H3K27me3 and only-H2AK121ub marked genes were significantly reduced in *bmi1abc*, increased in *clf28swn7*, and unaltered in *lhp1*, as previously reported^28^ (Fig. 3c, d). The fact that *clf28swn7* displayed increased H2AK121ub levels at both H2AK121ub/H3K27me3 and only-H2AK121ub marked genes (Fig. 3c, d) suggests an indirect consequence, since this effect was observed at only-H2AK121ub genes that are not CLF or SWN targets^41^. On the other hand, except for a few genes, we did not find significantly altered levels of H2AK121ub in *ring1ab* and *emf1-2* (Fig. 3c, d), which is consistent with a reduced but not eliminated RING1 activity and a low impact of EMF1 in H2AK121ub marking, respectively. All together, these data show that the levels of H2AK121ub are differentially affected in all these PcG mutants.

### H2AK121ub and EMF1 reduce chromatin accessibility at PcG targets

**Fig. 4:**
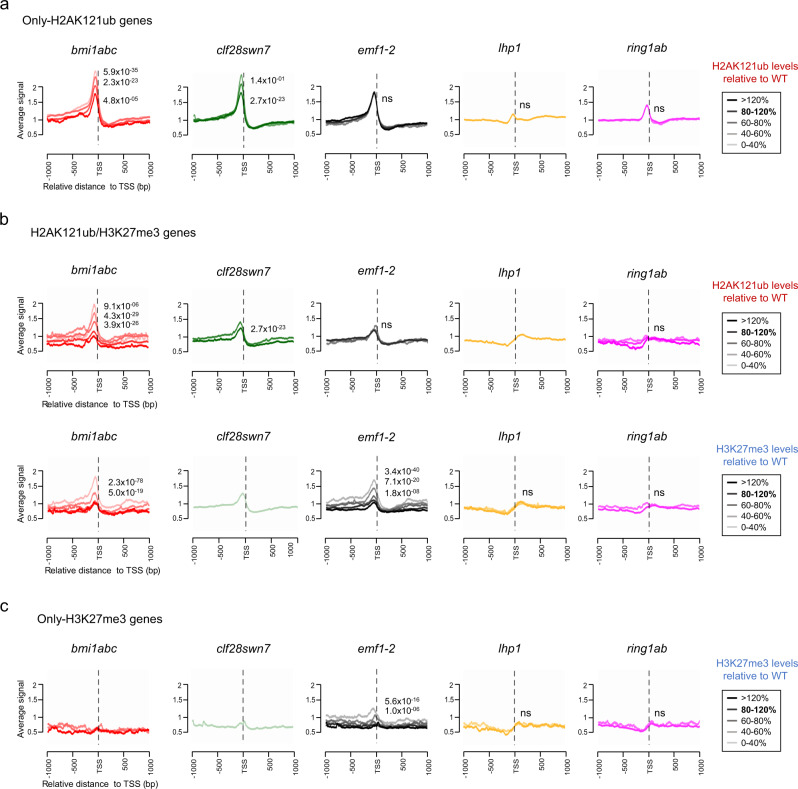
PRC1-mediated H2AK121ub and EMF1 in collaboration with PRC2 activity are required to reduce chromatin accessibility. **a** Accessibility profiles around the TSS of only-H2AK121ub-marked genes in the different mutants. Genes were classified into different groups depending on their levels of H2AK121ub marks relative to WT. The prolife of the different groups is indicated in a color increasing palette form lowest to highest H2AK121ub levels. **b** Accessibility profiles around the TSS of H2AK121ub/H3K27me3-marked genes in the different mutants, grouping the genes according to their levels of H2AK121ub marks (upper panels) or H3K27me3 (bottom panels) relative to WT. The prolife of the different gene groups is indicated in a color increasing palette form lowest to highest relative H2AK121ub or H3K27me3 levels. **c** Accessibility profiles around the TSS of only-H3K27me3-marked genes in the different mutants, grouping the genes according to their levels of H3K27me3 marks relative to WT. The prolife of the different gene groups is indicated in a color increasing palette form lowest to highest relative H3K27me3 levels. Gene groups with a very small number of genes were excluded as they produced noisy profiles (see Supplementary Data 3). In all cases (**a**–**c**), *p* values of significant differences between the profile of genes with WT-like levels (80–120%) and those of genes with different levels are indicated according to one-sided Mann–Whitney–Wilcoxon test.

Since we observed a generalized increase in chromatin accessibility in severe mutants associated with the embryonic phenotype, we investigated whether the loss of PcG marks actually plays a role in regulating chromatin accessibility. For this, we analyzed the accessibility profile around the TSS of only-H2AK121ub, H2AK121ub/H3K27me3 and only-H3K27me3 marked genes dividing the genes in groups according to their levels of H2AK121ub or H3K27me3 in the different mutants compared to WT (Fig. 4 and Supplementary Data 3). We first focused on only-H2AK121ub-marked genes (Fig. 4a). We found that decreasing levels of H2AK121ub led to a significant progressive increase in accessibility (Fig. 4a) whereas increasing levels led to reduced accessibility, supporting the association of H2AK121ub marks with a less accessible chromatin. However, we also found that expression levels of only-H2AK121ub-marked genes in WT, although not as high as in active genes lacking PcG marks (non-PcG targets), were higher than in H2AK121ub/H3K27me3, and only-H3K27me3-marked genes (Supplementary Fig. 7). Thus, as gene expression has been correlated with accessible chromatin^30,42^, these results suggest that H2AK121ub marks at only-H2AK121ub-marked genes associate with a less accessible although still permissive chromatin. Then, we analyzed H2AK121ub/H3K27me3-marked genes, which are generally repressed^28^. Since the incorporation of H3K27me3 at these genes is dependent on H2AK121ub^28^, their lower expression levels (Supplementary Fig. 7) suggests a role of H3K27me3 marks in further decreasing accessibility. Accordingly, we found that reduced levels of H2AK121ub in *bmi1abc* induced a significant loss of H3K27me3 and an increase in accessibility (Fig. 4b). Surprisingly, in *clf28swn7*, in which H3K27me3 is fully eliminated and H2AK121ub increases at many genes, accessibility was not increased as much as in *bmi1abc*, suggesting that either H2AK121ub and/or some other factor is preventing a gain of chromatin accessibility in this mutant. Importantly, although the levels of H2AK121ub were unaltered at H2AK121ub/H3K27me3-marked genes in *emf1-2*, genes with reduced levels of H3K27me3 in this mutant showed higher accessibility than in *clf28swn7*, indicating that EMF1, acting downstream H2AK121ub, contributes in reducing chromatin accessibility. Increased chromatin accessibility in *emf1-2* was also observed at only-H3K27me3-marked genes, which are not regulated by *bmi1abc* (Fig. 4c), supporting that EMF1 plays a role of in establishing an inaccessible chromatin.

We noted that the accessibility peak at only-H3K27me3-marked genes in *emf1-2* was smaller than that at H2AK121ub/H3K27me3 genes (Fig. 4b, c), suggesting that chromatin accessibility at only-H3K27me3 genes is less susceptible to a decrease in the levels of H3K27me3. On the other hand, although H3K27me3 levels were affected in *lhp1* and *ring1ab*, we did not find significant accessibility changes at any of the gene subsets.

### Accessibility increases in mutants independently of transcription

**Fig. 5:**
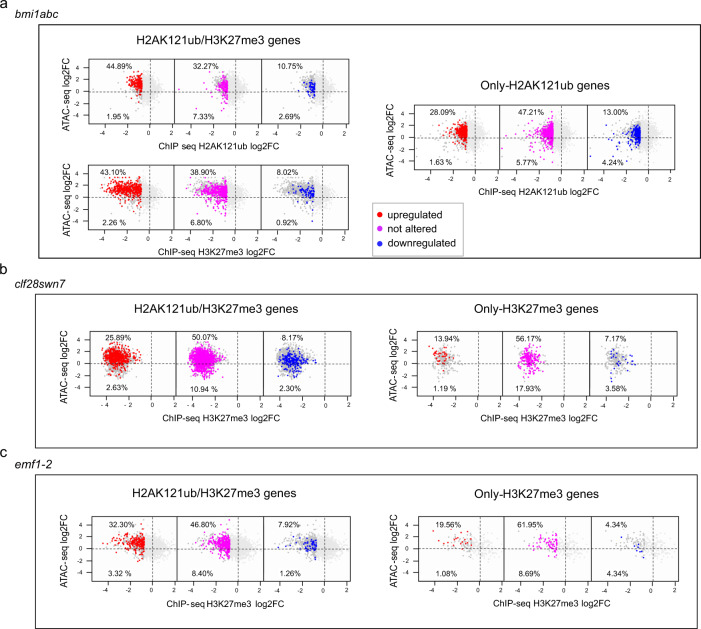
Increased chromatin accessibility in PcG mutants is not necessarily accompanied by gene expression. **a** Scatter plots showing the relationship between THS accessibility and marks levels (H2K121ub or H3K27me3) at H2AK121ub/H3K27me3 (left) and only-H2K121ub marked genes (right) in *bmi1abc* mutant. **b** Scatter plots showing the relationship between THS accessibility and H3K27me3 levels at H2AK121ub/H3K27me3 (left) and only-H3K27me3 marked genes (right) in *clf28swn7* mutant. **c** Scatter plots showing the relationship between THS accessibility and H3K27me3 levels at H2AK121ub/H3K27me3 (left) and only-H3K27me3 marked genes (right) in *emf1-2* mutant. In each panel, top numbers indicate the percentage of genes displaying altered or unaltered expression levels within the subset of genes showing less than 60% of WT H2K121ub or H3K27me3 (cutoff log2FC ≤ −0.74) and increased accessibility compared to WT. While bottom numbers indicate the same for the genes with less than 60% of WT H2K121ub or H3K27me3 and decreased accessibility compared to WT. The sum of top and bottom numbers from each panel represent 100% of the genes with less than 60% of WT H2K121ub or H3K27me3 and altered accessibility compared to WT. Upregulated expression is indicated in red, not altered expression in pink, and downregulated expression in blue. Source data are provided as a Source Data file.

Gene expression has been correlated with increased chromatin accessibility^31,37,42^. In line with this, we found that in WT the genes displaying the highest expression levels were the ones with highest chromatin accessibility (Supplementary Fig. 8). Hence, as loss of PcG function causes upregulation of a considerable number of PcG target and non-target genes^26,28,33,40,43^ (Supplementary Fig. 9a), PCA analysis of the transcriptome in the different mutants (Supplementary Fig. 9b) showed a similar clustering to the one observed with ATAC-seq data (Fig. 1a). However, it is also known that many PcG target genes do not become upregulated in PcG mutants despite losing PcG marks^26,28,33,40,43^. Therefore, we wondered whether the increased chromatin accessibility found at PcG targets in *bmi1abc*, *clf28swn7*, and *emf1-2* was a consequence of transcriptional upregulation of target genes or if it was caused by the loss of PcG function independently of the transcriptional state. To investigate this, we integrated accessibility, histone mark levels, and gene expression data in the different mutants. Since accessibility changes at only-H2AK121ub, H2AK121ub/H3K27me3, or only-H3K27me3 marked genes in mutants were evident at genes displaying less than 60% of WT H2AK121ub and/or H3K27me3 levels (Fig. 4), we analyzed whether or not the expression of the genes with less than 60% of WT marks (cutoff log2FC ≤ −0.74) was altered in *bmi1abc*, *clf28swn7*, and *emf1-2* (Fig. 5). Scatter plots in mutants representing accessibility versus histone mark levels confirmed that reduced levels of H2AK121ub and/or H3K27me3 led to increased chromatin accessibility (Fig. 5a–c and Supplementary Fig. 10). Interestingly, despite a high percentage of these genes were upregulated in mutants, we found a similar or even higher percentage of genes displaying unaltered expression levels. Remarkably, the percentage of genes with unaltered expression levels was particularly high in the subset of only-H3K27me3-marked genes (Fig. 5a–c). All together, these results indicate that the increased chromatin accessibility found at PcG targets is not caused by gene expression.

## Discussion

PcG complexes play central roles in eukaryotic gene regulation. However, despite these complexes have been proposed to define a closed chromatin conformation^44^, little is known about their role in regulating chromatin accessibility, especially in plants. Instead, PcG function has been extensively correlated to gene repression^44^.

While the association of PRC2 marking and gene repression is widely accepted in plants, PRC1-mediated H2AK121ub has been recently proposed to be associated with gene responsiveness^32^. Despite PRC1 activity is required to recruit PRC2 for H3K27me3 marking at many genes, there are also genes only marked with H2AK121ub or H3K27me3 (refs. ^28,32^). Interestingly, average expression levels of only-H2AK121ub genes are higher than H2AK121ub/H3K27me3 or only-H3K27me3 (ref. ^28^), which may argue against a repressive role of this modification. In line with this, a recent report proposed that H2AK121ub at H2AK121ub/H3K27me3-marked genes does not play a repressive role by itself, as despite it allows recruitment of PRC2, and thus gene repression, the removal of H2AK121ub at these genes seems to prevent gene reactivation by interfering with active H3K27me3 demethylation^32^. However, we found that the expression levels of only-H2AK121ub-marked genes were significantly lower than non-PcG active genes, supporting that this modification may play a role in modulating gene expression.

Nevertheless, although it is true that inaccessible chromatin is refractory to gene expression, chromatin accessibility by itself does not impose a transcriptional state^45^. Therefore, to understand PcG function it is fundamental to determine whether PRC1, PRC2, or their respective hallmarks play a role in regulating chromatin accessibility. Our data revealed that indeed PcG activities regulate chromatin accessibility. We found that reduced levels of H2AK121ub led to increased chromatin accessibility, whereas increased levels of this modification caused the opposite effect, supporting a role of this modification in regulating chromatin accessibility. It might be argued that the BMI1 proteins rather than H2AK121ub marks are the ones responsible for this effect. However, this role is supported by the fact that accessibility changes associated with the levels of H2AK121ub are observed in *clf28swn7*. In this mutant, the expression of *BMI1s* is not altered and the recruitment of the BMI1s should not be affected, as PRC1 recruitment is independent of PRC2. In any case, our data cannot rule out the possibility of a direct implication of BMI1 proteins in regulating chromatin accessibility instead of H2AK121ub.

**Fig. 6:**
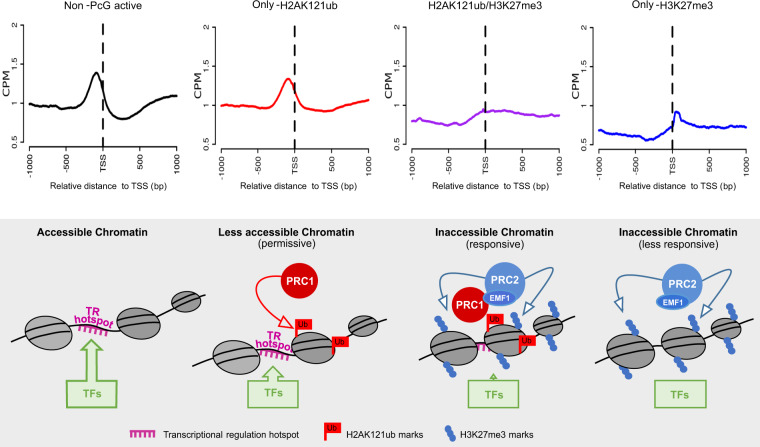
H2AK121ub, H3K27me3, and the combination of both marks define distinct chromatin accessibility states. Accessibility profiles around the TSS of non-PcG-marked active genes, only-H2AK121ub, H2AK121ub/H3K27m3, and only-H3K27me3 marked genes in WT (upper panels). Model proposed for the role of H2AK121ub and/or H3K27me3 marks in defining different chromatin states that control transcriptional responses to different degrees (bottom panels).

We also found that H2AK121ub associates with transcriptional regulation hotspots, which are sites enriched for the binding of a wide variety of TFs, and thus, required for transcriptional responses. Hence, we propose that H2AK121ub marks help to create a less accessible although still permissive chromatin at these regulatory hotspots (Fig. 6). This is consistent with the proposed hypothesis that H2AK121ub could provide a standby mode between activation and repression^32^.

In addition, our results indicate that chromatin accessibility can be further reduced by EMF1. This plant-specific protein has been shown to associate with PRC2 and to be required for H3K27me3 marking^19,21–25^. Accordingly, loss of EMF1 function affected H3K27me3 levels but not H2AK121ub. However, *emf1-2* ChIP-seq results showed that not all H3K27me3-marked genes display reduced levels. Furthermore, *emf1-2* phenotype suggests that EMF1 is dispensable for H3K27me3 marking at root tissue. Interestingly, a recent phylogenetic analysis of EMF1 showed that ancestral EMF1 gene duplicated twice resulting in three orthologous groups that, next to EMF1, contain three novel outparalogs^46^. This finding rises the possibility that these paralogs display EMF1-like functions in different tissues or in the regulation of different subsets of genes.

In any case, we found that the H3K27me3-marked genes that lose H3K27me3 in *emf1-2* displayed higher chromatin accessibility than *clf28swn7*, indicating that EMF1 acting upstream H3K27me3 marking plays an important role in regulating chromatin accessibility. Interestingly, EMF1 has the ability to in vitro mediate chromatin compaction^20^. Local and/or higher order chromatin compaction affects chromatin accessibility^45^. Furthermore, local chromatin compaction has been shown to precede the establishment of H3K27me3 in mouse embryonic stem cells^47^, which supports the connection between EMF1 and PRC2 activity. EMF1 ability to in vitro compact chromatin is shared with *Drosophila* PRC1 component Psc^20^, which combines BMI1 and EMF1 functions in a single polypeptide^48^, and with vertebrate Cbx2 (ref. ^13^), which is one of the Pc homologs^49^, indicating that, unlike in animals, EMF1 function in plants is associated to PRC2.

Based on our results, we also propose that chromatin at H2AK121ub/H3K27me3-marked genes associates with inaccessible but responsive chromatin, most likely due to the presence of H2AK121ub-marked transcriptional regulation hotspots, which may be important to allow gene reprogramming (Fig. 6). Conversely, only-H3K27me3-marked genes, which are generally not linked with these hotspots, are less responsive (Fig. 6), suggesting that the lack of H2AK121ub-marked transcriptional hotspots may prevent the ectopic expression of these genes.

In vertebrate, a recent report showed that despite PcG target promoters exhibit reduced chromatin accessibility, removal of PRC1, but not PRC2, causes reduced nucleosome occupancy and increase nucleosome spacing without affecting chromatin accessibility^16^. We found this result surprising, as one would have predicted that these effects would yield at least locally increased chromatin accessibility. In any case, under our experimental conditions and integrating chromatin accessibility, histone marks levels, and expression data in different PcG mutants, we were able to show that the loss of PcG activities in *Arabidopsis* leads to increased chromatin accessibility at PcG targets. Furthermore, our findings indicate that this increased chromatin accessibility is not caused by gene expression, which explains the lack of transcriptional activation of many PcG target genes that lose PcG marks in mutant plants^26,28,33,40,43^.

## Methods

### Plant materials and growth conditions

*Arabidopsis thaliana* WT Col-0, *bmi1abc*^8^, *clf28swn7* (ref. ^33^), *emf1-2* (ref. ^50^), *lhp1* (ref. ^51^), and *ring1ab*^34^ mutants were grown under long-day condition (16 h of light/8 h of dark) at 22 °C on MS agar plates containing 1% sucrose and 0.7% agar.

### ATAC-seq and data analysis

Nuclei isolation of ATAC-seq experiments were performed as previously described following the sucrose sedimentation protocol^52^. Briefly, nuclei from 10-day-old whole seedlings were separated from other cellular debris by passing the nuclei suspension through a sucrose cushion during centrifugation. After centrifugation, the contaminating organelles and debris are visible at the top of the tube and nuclei at the bottom. Next, 30,000 nuclei were counted and resuspended in 50 μL transposition reaction mix using the TruePrepTM DNA Library Prep Kit V2 for Illumina® (Vazyme, TD501). Reactions were incubated for 30 min at 37 °C before isolation DNA using the VAHTSTM DNA Clean Beads (Vazyme, N411). Two independent biological replicates were processed for next-generation sequencing library preparation. Libraries were prepared by PCR amplification using two index (i5 and i7) Illumina barcodes (Vazyme, TD202) with 13 cycles. Libraries were purified with a VAHTSTM DNA Clean Beads. ATAC libraries were sequenced on Illumina Hiseq-Xten PE150 by generating 2 × 150-bp paired-end reads.

We obtained around 45 million paired-end reads for each independent biological replicates of the different genotypes. Quality trimming and adapters removal were performed using Trimmomatic^53^. The organelle genomes were excluded from the TAIR10 *Arabidopsis* reference genome before reads were mapped using bowtie. PCR duplicates were discarded from the mapped reads using Picard (Picard Toolkit, 2019, Broad Institute, GitHub Repository. http://broadinstitute.github.io/picard/; Broad Institute). Regions with an artifactual massive amount of unique mapped reads were identified. Specifically, 91 regions were collected into a list of blacklisted genomic regions (Supplementary Data 4). In order to prevent interference with subsequent analysis steps, reads mapped to the blacklisted genomic regions were eliminated using samtools. Next, THSs were identified by performing a peak calling analysis using MACS2 with parameters–nomodel–shift -100–extsize 200 -q 0.05. For each genotype, genotype-specific THSs were determined by intersecting the THSs found on each individual replicate using bedtools. A final set of consensus THSs was generated by merging the THSs identified in each genotype. Using different deepTools functions we generated bigwig files storing the genome-wide accessibility signal measured as CPM for the different genotype replicates. Average accessibility profiles for the different genotypes were generated using the bioconductor R package ChIPpeakAnno. An accessibility signal matrix was generated where rows represented THSs and columns genotype replicates. For each THS, its average accessibility signal was computed for each sample. Rows corresponding to THSs exhibiting a maximum signal level below the 5% lower percentile of the signal distribution, approximately 3 CPM were filtered out in order to reduce noise in our analysis. After log2 transformation of the filtered matrix, the bioconductor R package limma was used to identify significantly reduced or increased accessibility levels in the different mutants when compared to WT-like levels (80–120% levels). The statistical significance of differences in accessibility around TSS were assessed using the non-parametric Mann–Whitney–Wilcoxon test implemented in the Wilcox.test function in R. Regression analysis was performed using the *lm* (linear model) function in R. The overall significance of the regression analysis was carried out using the *F*-test.

The bioconductor R package ChipseekeR^54^ was used to study the location of THS around the TSS. The percentages of consensus THSs located nearby histone marks were determined using different functions from deepTools.

Transcription factor-binding sites (TFBS) have been determined in this study by reanalyzing in a uniform manner 100 independent ChIP-seq datasets previously published covering most TF families in *Arabidopsis thaliana* (see Supplementary Data 2). The TFBS enrichment at the THS was estimated using Monte Carlo simulations. First, we computed the median number of TFBS overlapping with our consensus THSs using deepTools. Then, we generated 10^4^ sets of random genomic regions using a custom R script. These random sets were composed of as many genomic regions as consensus THSs. Moreover, the random regions have the same length as our THSs. For each one of the randomly generated sets we computed the median number of TFBS overlapping with the corresponding random genomic regions. By comparing these random values to the actual number of TFBS in our consensus THSs we estimated the corresponding *p* value and enrichment.

### ChIP-seq and data analysis

H2AK121ub and H3K27me3 ChIP-seq data of *bmi1abc*, *clf28swn7*, and WT at 7 DAG, and H2AK121ub data of *lhp1* at 7 DAG were previously generated^28^. H2AK121ub and H3K27me3 ChIP-seq data of WT, *ring1ab*, *emf1-2*, and H3K27me3 data of *lhp1* at 10 DAG were generated for this work.

ChIP-seq experiments were performed as previously described^55^. In brief, whole seedlings were fixed in 1% formaldehyde. Chromatin was extracted from fixed tissue and fragmented using a Bioruptor® Pico (Diagenode) in fragments of 200–500 bp. The sheared chromatin was immunoprecipitated overnight using the following antibodies: Anti-H2Aub (Cell Signaling Technology, 8240S, dilution 1:100) and anti-H3K27me3 (Millipore, 07-449, dilution 1:300). Immunocomplexes were captures using Protein A Sepharose beads CL-4B (GE Healthcare). After washing the Protein-A beads, chromatin was eluted and the crosslinking was reversed overnight at 65 °C. The DNA from the immunoprecipitated chromatin was treated with RNase and proteinase K and purified by phenol–chloroform extraction followed by ethanol precipitation. For ChIP-seq, two immunoprecipitations from independent biological replicates were processed for next-generation sequencing library preparation. All libraries were prepared with end repair, A-tailing and ligation of Illumina-compatible adapters using the Ovation® Ultralow Library Systems (NuGEN). The ligated product was amplified with 14 cycles of PCR. DNA of a size range between 200 and 600 bp was purified from an agarose gel. Amplification was confirmed by testing an aliquot of the library before and after amplification by qPCR. Libraries were sequenced on Illumina Hiseq-Xten PE150 by generating 2 ×  150-bp paired-end reads.

Each sample for the different genotypes generated approximately 12 million paired-end reads. Bowtie^56^ was used to map reads to the TAIR10 *Arabidopsis* reference genome. The tool boxes for the analysis of high-throughput sequencing data deepTools^57^ and samtools^58^ were used to generate bigwig files storing H3K27me3 and H2AK121ub genome-wide signal levels measured as CPM (counts per million). Specifically, the function bamCoverage was used to compute normalized signal levels using the parameter –normalizeUsing CPM. Mark peaks were detected using MACS2 (ref. ^59^) with default parameters for each individual replicate of the WT genotype. Specifically, the function bamCoverage was used to compute normalized signal levels using the parameter –normalizeUsing CPM. Mark peaks were detected using MACS2 (ref. ^59^) with default parameters for each individual replicate of the WT genotype. A specific input for each of the two datasets was considered, namely an input for the previously published data corresponding to 7 DAG and another specific input for the data generated in this study from 10 DAG seedlings. The final set of mark peaks were defined as the intersection of the ones detected for each replicate and the combination obtained from the 7 DAG and 10 DAG WT data using bedtools^60^. Target genes were associated to mark peaks using the bioconductor R package ChIPpeakAnno^61^ when an overlap was detected with either the gene body or promoter defined as the region 750 bp upstream from the TSS. A matrix was constructed were rows represented marked genes and columns the different genotype samples. For each marked gene a signal level in each one of the genotype samples under study was computed as the average signal across the corresponding histone mark peak. In order to remove noisy peaks, we filtered out genes with signals below 3 CPM, approximately corresponding to the 5% lower percentile of the signal distribution. After log2 transformation of the filtered matrix the bioconductor R package limma^62^ was used to identify significantly reduced or increased histone mark signal levels in the different mutants when compared to the WT.

Metagene plots were generated with a custom R script available from our github repository (10.5281/zenodo.4304639)^69^. The statistical significance of differences in metagene plots were assessed using the non-parametric Mann–Whitney–Wilcoxon test implemented in the Wilcox.test funcion in R. Similarly, the identification of binding sites for the TFs under analysis was performed using bowtie for read mapping and macs2 for peak calling.

### ChIP-reChIP-qPCR analysis

ChIP-reChIP experiments were performed as previously described^63^. In brief, 10-day-old WT seedlings were fixed in 1% formaldehyde. Chromatin was extracted from fixed tissue and fragmented using a Bioruptor® Pico (Diagenode) in fragments of 500–1000 bp. ChIP was first performed with anti-H3K27me3 (Millipore, 07-449, dilution 1:300). Immunocomplexes were captures using Protein A Sepharose beads CL-4B (GE Healthcare). After washing the Protein-A beads, one fraction was processed as in a conventional ChIP assay in order to revert the crosslinking and purify the DNA, and another fraction was used to elute the immunoprecipitated protein–DNA complexes in Re-ChIP elution buffer (2 mM EDTA, 500 mM NaCl, 0.1% SDS, 1% NP40) by incubation for 30 min at 37 °C. The eluate was diluted 20 times with ChIP dilution buffer supplemented with 50 μg of BSA and protease inhibitor. Then, the second ChIP was performed with anti-H2Aub (Cell Signaling Technology, 8240S, dilution 1:100). Immunocomplexes were captures using Protein A Sepharose beads CL-4B (GE Healthcare). After washing the Protein-A beads, chromatin was eluted, de-crosslinked, and the DNA was extracted. The DNA obtained in the two sequential ChIPs were used as PCR template to amplify *PLT5*, *SOC1*, and *AG* using specific primers (Supplementary Table 1).

### RNA-seq and data analysis

WT at 7 DAG RNA-seq data of *bmi1abc* have been previously published^28^. RNA-seq data of WT and the remaining mutants at 10 DAG were generated for this work.

Total RNA was extracted using E.Z.N.A.^®^ Plant RNA Kit (Omega, R6827-01) from 10-day-old whole seedlings following the manufacturer´s instructions. Three independent biological replicates were processed for next-generation sequencing library preparation. Libraries were prepared using 1 μg of total RNA with the VAHTSTM Total RNA-seq (H/M/R) Library Prep Kit for Illumina® (Vazyme, NR603) according to the manufacturer’s instructions. Libraries were sequenced on Illumina Hiseq-Xten PE150 by generating 2 × 150-bp paired-end reads.

Each independent biological replicate of the different genotypes generated approximately 20 million paired-end reads. Quality control was performed using FASTQC. Read mapping to the TAIR10 *Arabidopsis* reference genome was carried out using HISAT2 (ref. ^64^). Transcript assembly and gene expression estimation measured as FPKM (fragments per kilobase of exon and million of mapped reads) were computed using Stringtie^65^ and the bioconductor R package ballgown^66^. Differential gene expression analysis was performed using the bioconductor R package limma^62^. Specifically, a log2 fold-change of ±1 and a *p* value of 0.05 was used to determine activated, repressed, and unaltered genes. GO enrichment analysis was performed over the different gene sets of interest using the bioconductor R package clusterProfiler^67^. PCA was carried out using the R package FactoMineR v2.3 (ref. ^68^).

### Reporting summary

Further information on research design is available in the Nature Research Reporting Summary linked to this article.

## Supplementary information

Supplementary Information

Peer Review File

Reporting Summary

Description of Additional Supplementary Files

Supplementary Data 1

Supplementary Data 2

Supplementary Data 3

Supplementary Data 4

## Data Availability

Data supporting the findings of this work are available within the paper and its Supplementary Information files. A reporting summary for this article is available as a Supplementary Information file. The datasets and plant materials generated and analyzed during the current study are available from the corresponding author upon request. ATAC-seq, ChIP-seq, and RNA-seq datasets generated in this study have been deposited in the Gene Expression Omnibus (GEO) under accession GSE155378. Previously generated ChIP-seq and RNA-seq data are under accession GSE89358.  Source data are provided with this paper.

## Source data

Source Data

## Code availability

Several custom R scripts that were used to generate signal profiles have been deposited in the GitHub repository (10.5281/zenodo.4304639)^69^.
