# Supplementary material for: H2AK121ub in Arabidopsis associates with a less accessible chromatin state at transcriptional regulation hotspots
Source: Nat Commun. 2021 Jan 12;12:315. doi: 10.1038/s41467-020-20614-1 (PMC7804394; doi:10.1038/s41467-020-20614-1)
Supplement: Supplementary file 1 — Supplementary Information [file 41467_2020_20614_MOESM1_ESM.pdf]

H2AK121ub in *Arabidopsis* associates with a less accessible chromatin state at transcriptional regulation hotspots

Yin and Romero-Campero *et al.*

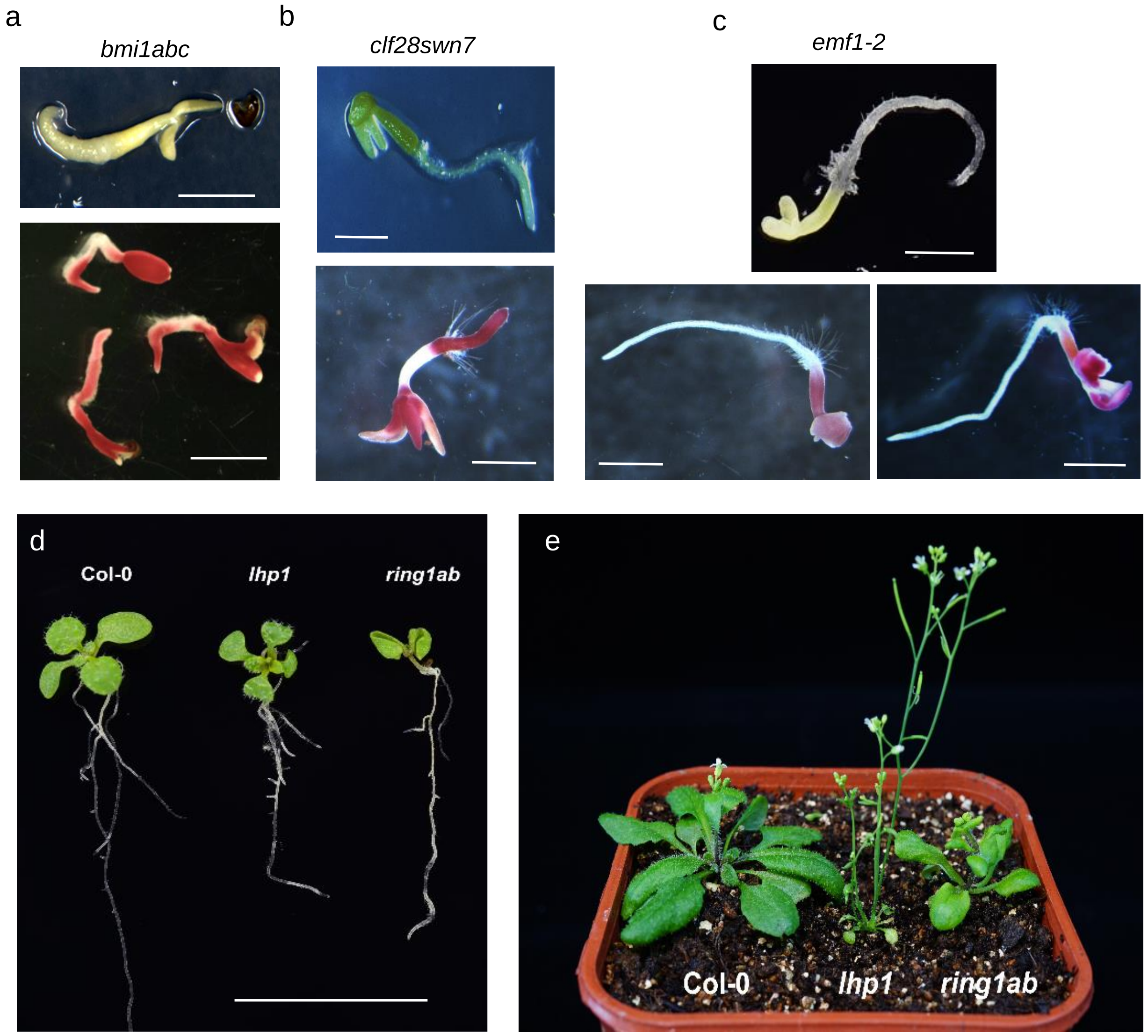

Supplementary Figure 1. PcG mutant phenotypes. *bmi1abc* and *clf28swn7* show a swollen pickle-like primary root that accumulates triacylglycerol as indicated by Fat Red staining, while *emf1-2* has a shorter but WT-like root that does not accumulate lipids. **(a)** Phenotype of *bmi1abc* at 10 DAG (upper picture). Fat Red staining of *bmi1abc* mutants showing accumulation of triacylglycerol both in cotyledons and root (bottom picture). **(b)** Phenotype of *clf28swn7* at 10 DAG (upper picture). Fat Red staining of *clf28swn7* mutant showing accumulation of triacylglycerol in aerial and root tissues (bottom picture). **(c)** Phenotype of *emf1-2* at 10 DAG (upper pictures). Fat Red staining of *emf1-2* mutants showing accumulation of triacylglycerol only in the aerial part of the seedling (bottom pictures). Bars indicate 2 cm. **(d,e)** *lhp1* and *ring1ab* weak mutants are able to develop vegetative and floral organs in spite of several developmental alterations. WT Col-0, *lhp1* and *ring1ab* at 10 DAG (d)**.** Three weeks old WT Col-0, *lhp1* and *ring1ab* mutants growing on soil (e). Bars indicate 1 cm.

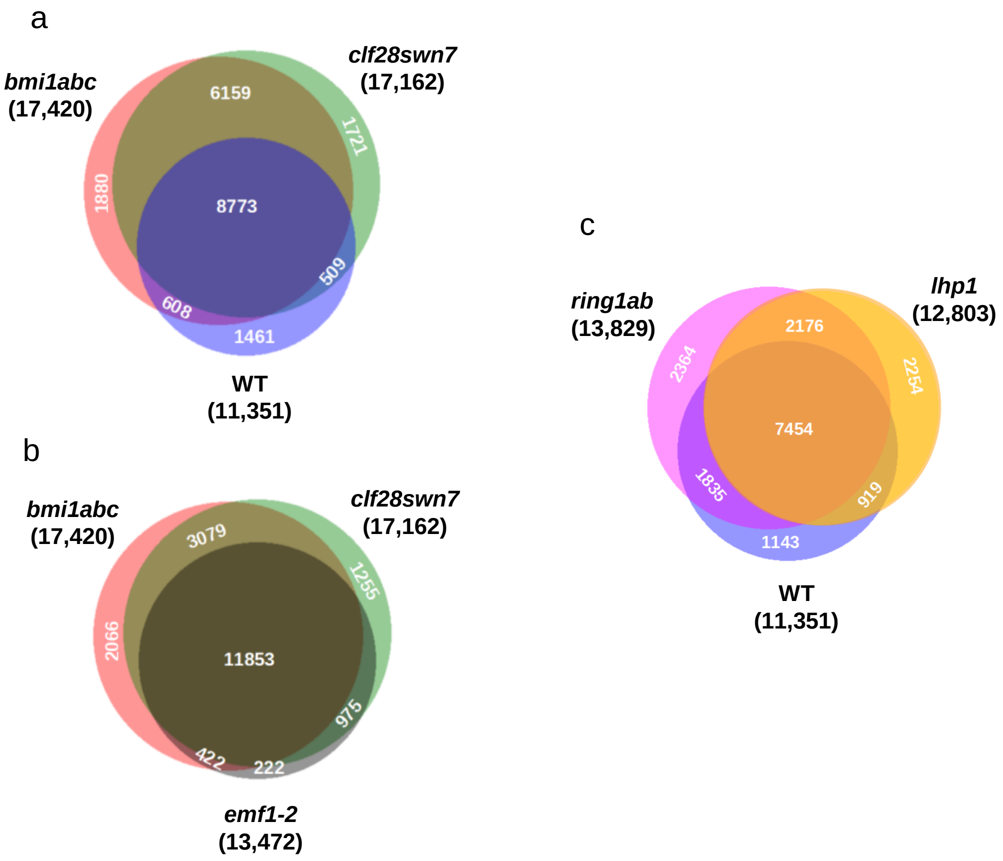

Supplementary Figure 2. Comparison of THSs found in the different genotypes. (a) Venn diagram showing common and unique THSs between *bmi1abc*, *clf28swn7* and WT at 10 DAG. **(b)** Venn diagram showing common and unique THSs between *bmi1abc*, *clf28swn7* and *emf1-2* at 10 DAG. **(c)** Venn diagram showing common and unique THSs between *ring1ab*, *lhp1* and WT at 10 DAG. The number of THSs found at the different genotypes is indicated in parenthesis.

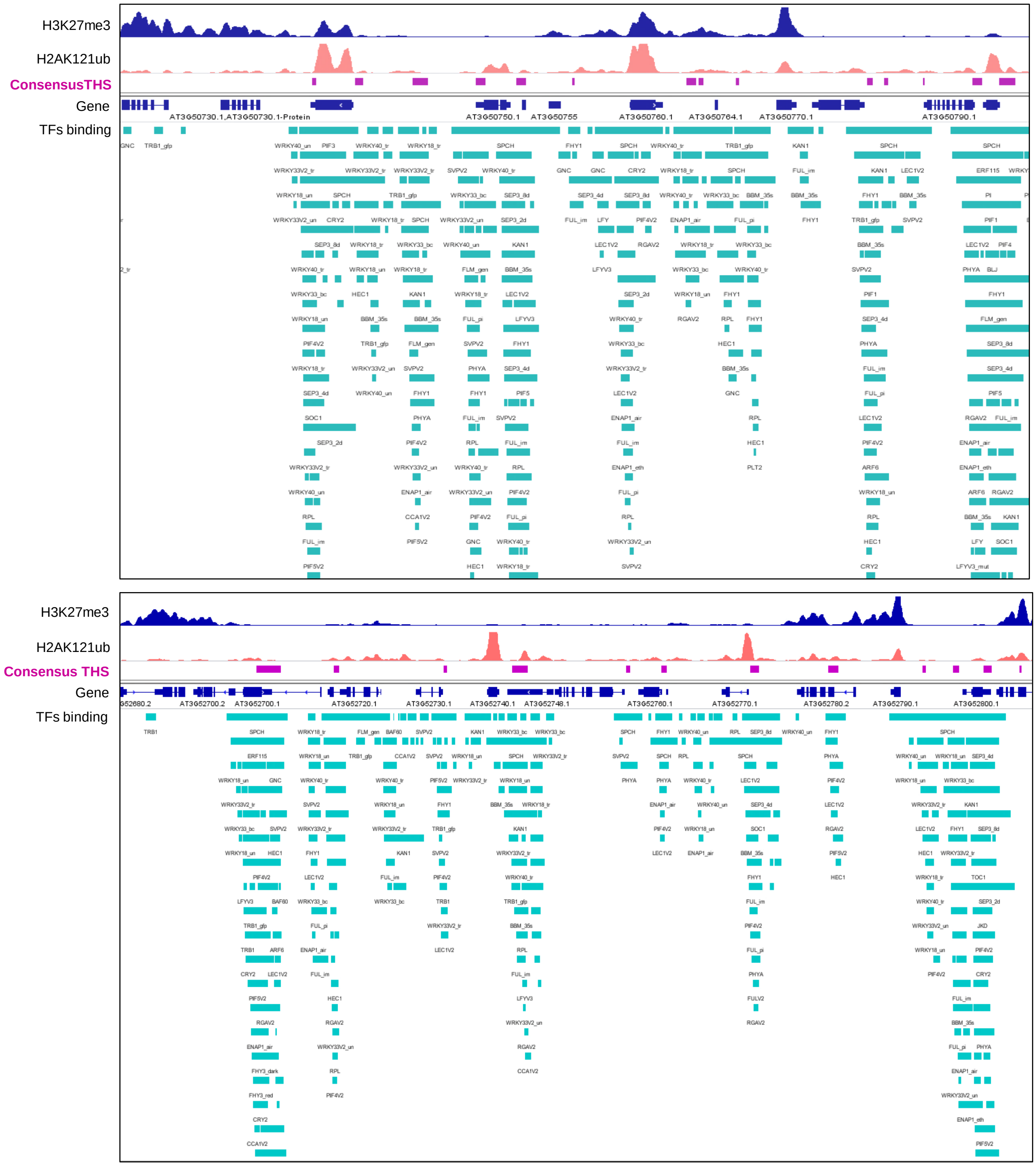

Supplementary Figure 3. H2AK121ub hallmarks transcriptional regulation hotspots. Screen shots showing distribution of H3K27me3, H2AK121ub in WT, consensus THSs and TFs binding sites. H3K27me3 peaks are indicated in blue, H2AK121ub peaks in red, consensus THSs regions in magenta, gene regions in blue and TFs binding sites in greenish blue. The name of the TFs is included (see Supplementary Data 2).

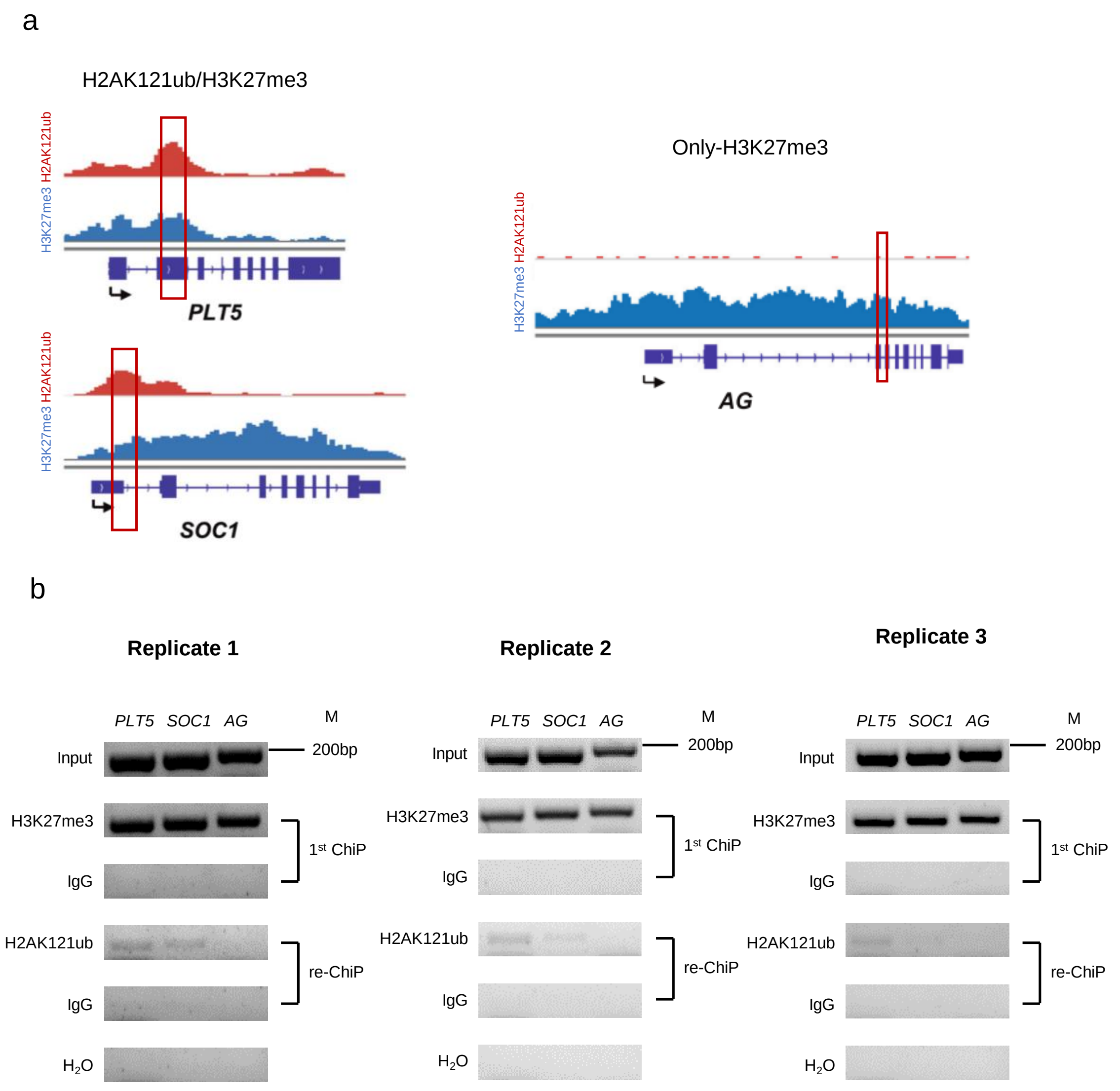

Supplementary Figure 4. ChIP-reChIP experiments using anti-H3K27me3 and anti-H2AK121ub antibodies. (a) Screen shots showing distribution of H3K27me3 and H2AK121ub at *PLETHORA 5* (*PLT5*), *SUPPRESSOR OF OVEREXPRESSION OF CO 1* (*SOC1*) and *AGAMOUS* (*AG*). H2AK121ub peaks are indicated in red, H3K27me3 peaks in blue and gene regions in dark blue. Red boxes indicate the regions amplified by ChIP-reChIP-qPCR using specific primers (see Methods). **(b)** ChIP-reChIP-qPCR results of three independent biological replicates. Non-specific IgG controls in 1st ChIP and re-ChIP as well as PCR water (H_2O) control are included. Approximated size of the amplified fragments is indicated according to a molecular marker (M). Uncropped gel images are provided as a Source Data file.

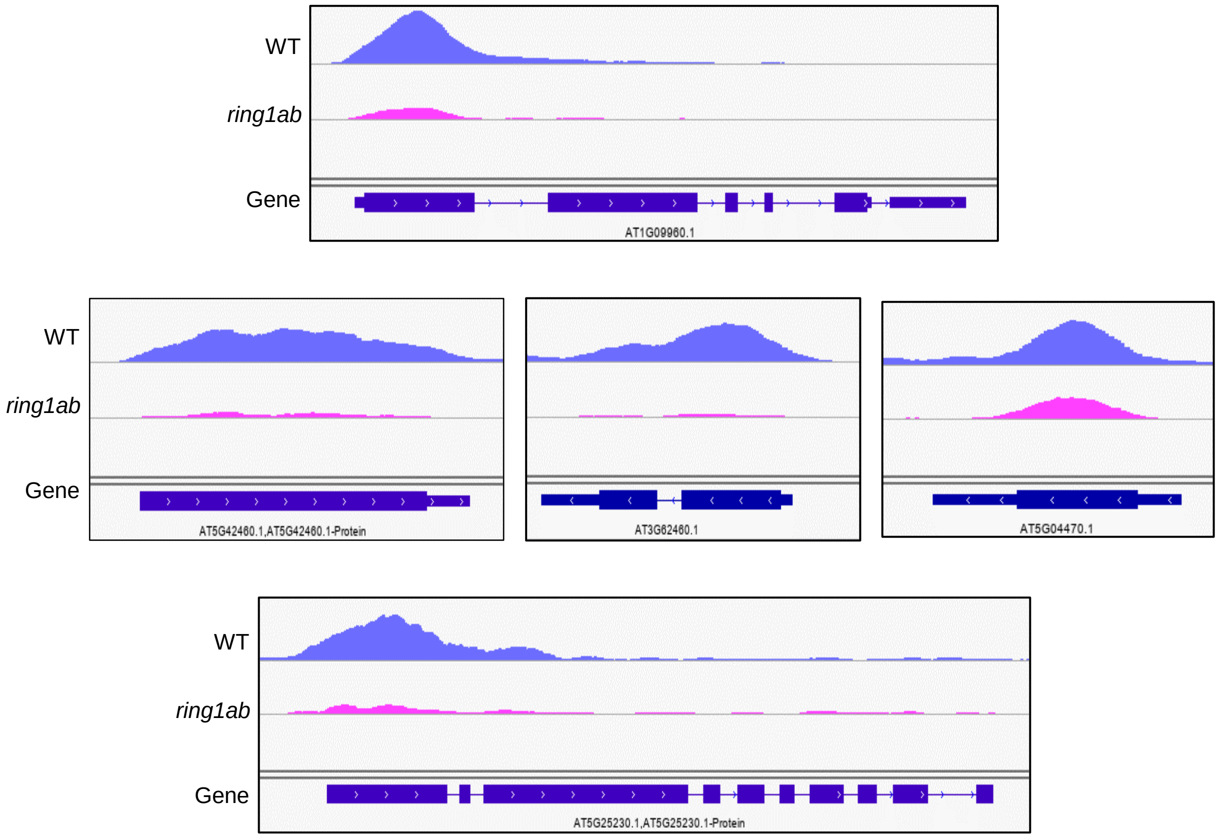

Supplementary Figure 5. Genes displaying strongly decreased levels of H3K27me3 in *ring1ab* . Screenshots showing the levels of H3K27me3 at selected genes in *ring1ab* and WT**.**

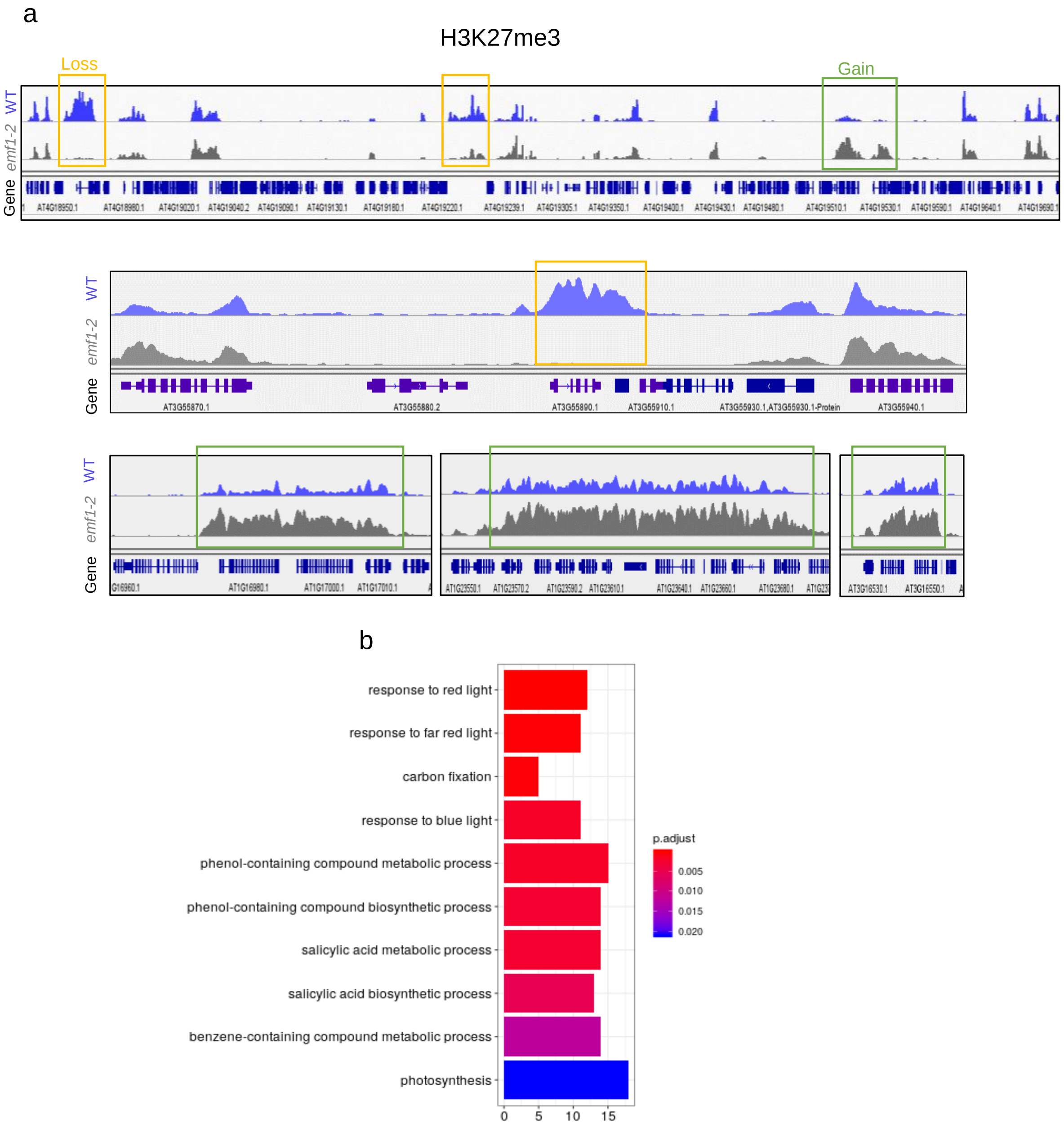

Supplementary Figure 6. Genes with affected levels of H3K27me3 in *emf1-2* mutants. (a) Screenshots showing the levels of H3K27me3 at different genes in *emf1-2* and WT. In yellow are indicated regions with reduced levels in *emf1-2* and in green with increased levels. **(b)** GO enrichment analysis of genes with increased levels of H3K27me3 in *emf1-2.* The hypergeometric test was used to determine significant enrichment. The analysis shows an enrichment of genes involved in photosynthesis and response to light, which are normally repressed by PcG in root.

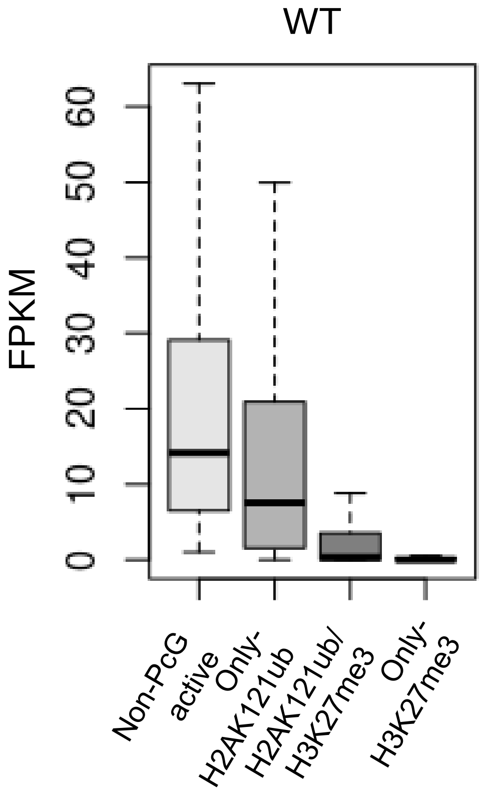

Supplementary Figure 7. Only-H2AK121ub genes display higher average expression levels that H2AK121ub/H3K27me3 or only-H3K27me3 marked genes in WT. Box plots showing expression levels of non-PcG marked active genes, only-H2AK121ub, H2AK121ub/H3K27me3 and only-H3K27me3 marked genes in WT seedlings at 10 DAG. The statistical significance of the differences between only-H2AK121ub and the other gene sets was assessed using the non-parametric one-sided Mann-Whitney-Wilcoxon test resulting in p-values $< 2.2 \times 10^{-16}$ in all cases. Data were generated from n=3 biological independent RNA-seq samples. Box plots show the median (middle line), upper and lower quartiles (boxes) and minimum and maximum values (whiskers).

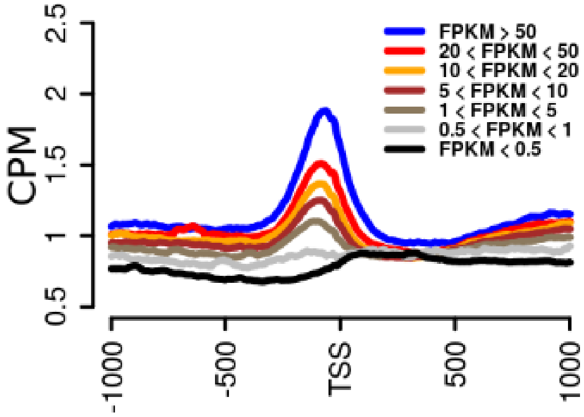

Supplementary Figure 8. Gene expression is correlated with chromatin accessibility. Average accessibility profile at the region surrounding the TSS of genes displaying different expression levels (FPKM) in WT at 10 DAG.

a

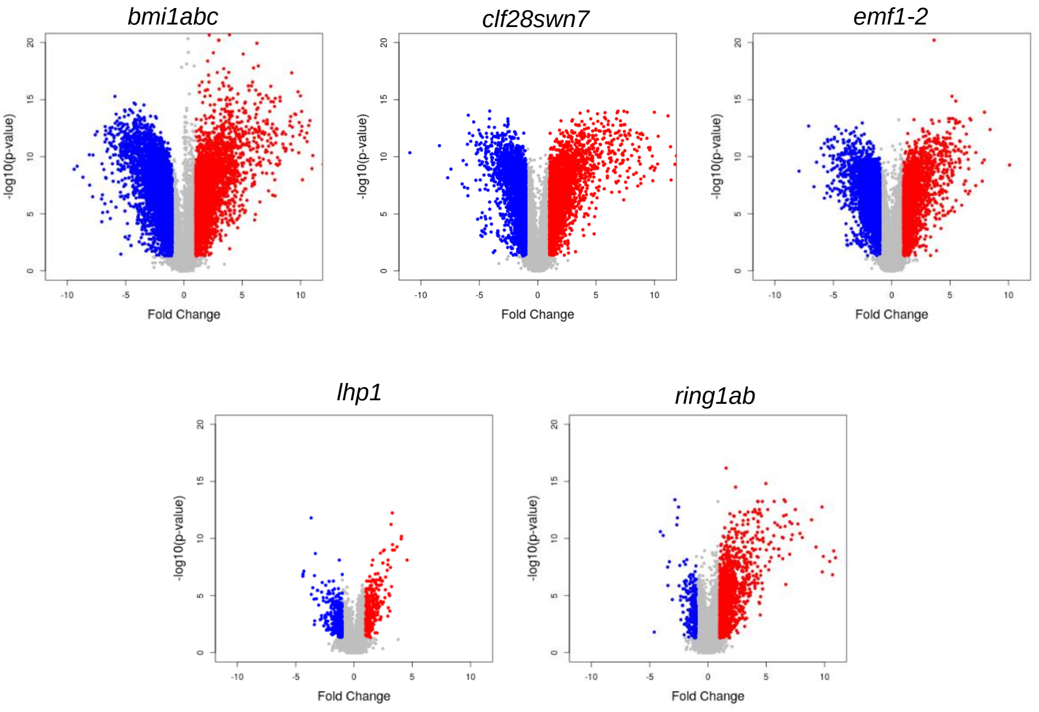

b

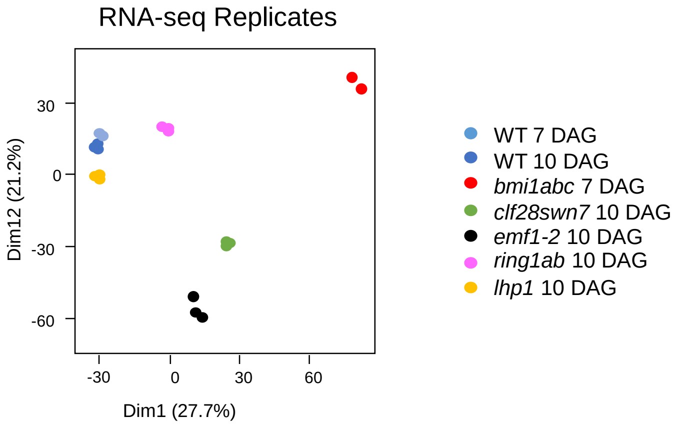

Supplementary Figure 9. Differentially expressed genes in the different mutants. (a) Volcano plots showing downregulated (blue) and upregulated (red) genes in *bmi1abc* at 7 days after germination (DAG), and *clf28swn7*, *emf1-2*, *lhp1* and *ring1ab* mutants at 10 DAG compared to WT at 7 DAG and 10 DAG, respectively. Differentially expressed genes were determined based on a Log2(FC) +/-1 and a q-value of 0.05 computed according to a moderated t-statistics with Benjamin-Hochberg multiple testing correction. (b) PCA analysis of WT at 7 DAG, WT at 10 DAG, *bmi1abc* at 7 DAG, *clf28swn7* at 10 DAG, *emf1-2* at 10 DAG, *lhp1* at 10 DAG and *ring1ab* at 10 DAG RNA-seq replicates.

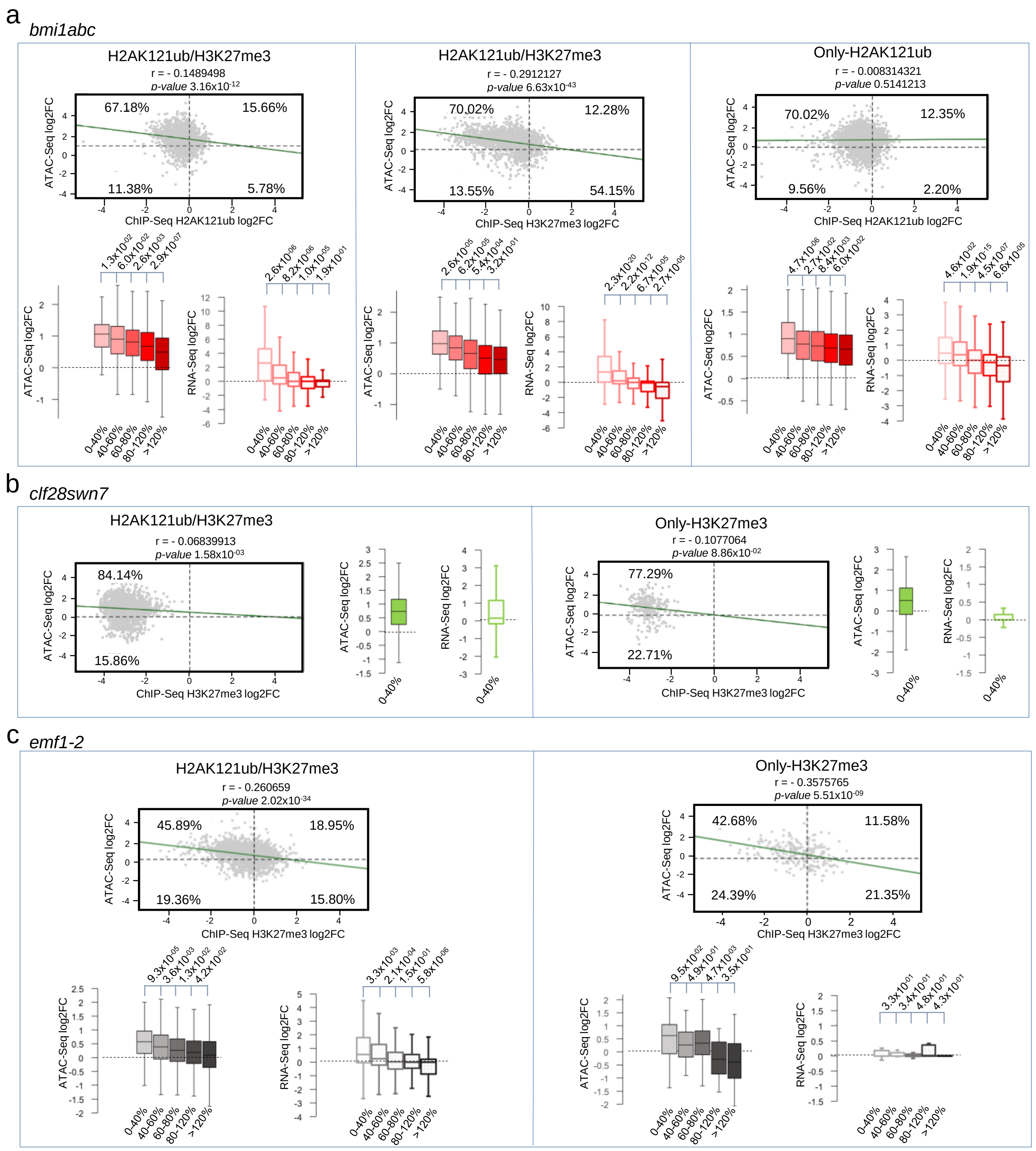

Supplementary Figure 10. Reduced levels of H2AK121ub and/or H3K27me3 led to increased chromatin accessibility. Scatter plots showing the relationship between accessibility at THS and levels of H2K121ub or H3K27me3 marks at PcG marked genes *in bmi1abc* **(a)**, *clf28swn7* **(b)** and *emf1-1* **(c)**. In each case, the correlation coefficient (r) and p-value according to F test are indicated. Percentage of genes with increased or decreased levels of accessibility and histone marks are indicated. Also, box plots showing accessibility (left) and expression (right) changes in genes grouped according to their levels of H2AK121ub or H3K27me3 marks relative to WT (0-40%, 40-60%, 60-80%, 80-120% and >120%) are included in each panel. Median (middle line), upper and lower quartiles (boxes) and minimum and maximum values (whiskers) are indicated. P-value of differences between groups are indicated according to one-sided t-student test. Data were generated from n=2 biological independent ATAC-seq samples and n=3 biological independent RNA-seq samples. Note that except for the case of only-H2AK121ub marked genes, in all cases there is weak but significant negative correlation between the levels of histone marks and accessibility. Nevertheless, comparison of accessibility changes among the different only-H2AK121ub groups in *bmi1abc* (third upper panel box plots), also indicates a negative relationship between H2AK121ub levels and accessibility. The apparent lack of correlation is most probably due to the fact that the genes that do not lose marks (80-120% and >120%) in this subset of genes show a higher degree of accessibility than those genes in the other subsets of PcG marked genes, thus, reducing the strength of the relationship. Source data are provided as a Source Data file.

Supplementary Table 1. ChIP-PCR primers used to amplify *PLT5*, *SOC1*, and *AG* in ChIP-reChIP experiments.

Primer name	Sequence
**PLT5-RE-F**	5’-GCGTTGTCTTCTCTTCCGAC-3’
**PLT5-RE-R**	5’-ACAGCTAGAGGATTCGGTTGT-3’
**SOC1-RE-F**	5’- ATGAATTCGCCAGCTCCAAG-3’
**SOC1-RE-R**	5’- ACACCTCTCAAAGCAAACGT-3’
**AG-RE-F**	5’- TGTAAAAGGGACTATTGAGAG-3’
**AG-RE-R**	5’- GGCTGATTCTTGTTGATAATA-3’
