# Supplementary material for: H2AK121ub in Arabidopsis associates with a less accessible chromatin state at transcriptional regulation hotspots
Source: Nat Commun. 2021 Jan 12;12:315. doi: 10.1038/s41467-020-20614-1 (PMC7804394; doi:10.1038/s41467-020-20614-1)
Supplement: Supplementary file 3 — Reporting Summary [file 41467_2020_20614_MOESM3_ESM.pdf]

nature research

Corresponding author(s): Myriam Calonje
Yue Zhou
Francisco J Romero-Campero

Last updated by author(s): Dec 3, 2020

Reporting Summary

Nature Research wishes to improve the reproducibility of the work that we publish. This form provides structure for consistency and transparency in reporting. For further information on Nature Research policies, see our Editorial Policies and the Editorial Policy Checklist.

Statistics

For all statistical analyses, confirm that the following items are present in the figure legend, table legend, main text, or Methods section.

n/a	Confirmed	
☐	☒	The exact sample size (*n*) for each experimental group/condition, given as a discrete number and unit of measurement
☐	☒	A statement on whether measurements were taken from distinct samples or whether the same sample was measured repeatedly
☐	☒	The statistical test(s) used AND whether they are one- or two-sided<br>*Only common tests should be described solely by name; describe more complex techniques in the Methods section.*
☐	☒	A description of all covariates tested
☐	☒	A description of any assumptions or corrections, such as tests of normality and adjustment for multiple comparisons
☐	☒	A full description of the statistical parameters including central tendency (e.g. means) or other basic estimates (e.g. regression coefficient) AND variation (e.g. standard deviation) or associated estimates of uncertainty (e.g. confidence intervals)
☐	☒	For null hypothesis testing, the test statistic (e.g. *F*, *t*, *r*) with confidence intervals, effect sizes, degrees of freedom and *P* value noted<br>*Give P values as exact values whenever suitable.*
☐	☒	For Bayesian analysis, information on the choice of priors and Markov chain Monte Carlo settings
☐	☒	For hierarchical and complex designs, identification of the appropriate level for tests and full reporting of outcomes
☐	☒	Estimates of effect sizes (e.g. Cohen's *d*, Pearson's *r*), indicating how they were calculated

Our web collection on statistics for biologists contains articles on many of the points above.

Software and code

Policy information about availability of computer code

Data collection	No software was used for data collection.
Data analysis	Quality control was performed using FASTQC v0.11.9 and Trimmomatic v0.39. Read mapping to the reference genome was carried out using HISAT2 v2.2.0 for RNA-seq data and bowtie2 v2.4.1 for ChIP-seq and ATAC-seq data. Transcript assembly and gene expression estimation was performed using StringTie v2.1.1. Differential Gene Expression analysis were carried out with the Bioconductor R packages Ballgown v2.20.0 and limma v3.44.3. GO term enrichment analysis were performed using the Bioconductor R packages clusterProfiler v3.16.0 and org.At.tair.db 3.11.4. Peakcalling and THS identification were carried out using MACS2 v2.2.6. BED file intersection and union were performed using bedtools v2.28.0. BAM file conversion was carried out with samtools v1.10. PCR Duplicates from ChIP-seq and ATAC-seq data were removed using Picard v2.21.0. BW files were generated using deepTools v3.3.2. Peaks and THS annotation were performed using the Bioconductor R packages ChIPpeakAnno v3.22.2 and ChIPSeeker v1.24.0. Several custom R scripts were used to generated signal profiles that are deposited in the GitHub repository https://github.com/fran-romero-campero/Rmetageneplots. Principal components analysis was performed using the R package FactoMiner v2.3.

For manuscripts utilizing custom algorithms or software that are central to the research but not yet described in published literature, software must be made available to editors and reviewers. We strongly encourage code deposition in a community repository (e.g. GitHub). See the Nature Research guidelines for submitting code & software for further information.

Data

Policy information about availability of data

All manuscripts must include a data availability statement. This statement should provide the following information, where applicable:

- Accession codes, unique identifiers, or web links for publicly available datasets
- A list of figures that have associated raw data
- A description of any restrictions on data availability

Data supporting the findings of this work are available within the paper and its Supplementary Information files. The datasets and plant materials generated and analyzed during the current study are available from the corresponding author upon request. ATAC-seq, ChIP-seq and RNA-seq data sets generated in this study have been deposited in the Gene Expression Omnibus (GEO) under accession GSE155378 (https://www.ncbi.nlm.nih.gov/geo/query/acc.cgi?acc=GSE155378). Previously generated ChIP-seq and RNA-seq data are under accession GSE89358. The source data underlying Figure 3, Figures 5, as well as Supplementary Figures 4, and 10 are provided as a Source Data file.

Field-specific reporting

Please select the one below that is the best fit for your research. If you are not sure, read the appropriate sections before making your selection.

- [x] Life sciences
- [ ] Behavioural & social sciences
- [ ] Ecological, evolutionary & environmental sciences

For a reference copy of the document with all sections, see nature.com/documents/nr-reporting-summary-flat.pdf

Life sciences study design

All studies must disclose on these points even when the disclosure is negative.

Sample size	We used 2 grams of 10-day-old seedlings from the different genotypes for ChIP-seq and ATAC-seq based on the published method, and 0.1 gram of 10-day-old seedlings for RNA-seq based on the RNA extraction kit instruction. We analyzed two biological replicates of each genotype for ATAC-seq and ChIP-seq and three for RNA-seq, which according to ATAC-seq Guidelines of Harvard FAS Informatics, and ChIP-seq and RNA-seq guidelines and practices of the ENCODE project are appropiated (see: https://informatics.fas.harvard.edu/atac-seq-guidelines.html https://www.encodeproject.org/documents/ceb172ef-7474-4cd6-bfd2-5e8e6e38592e/@@download/attachment/ChIP-seq_ENCODE3_v3.0.pdf https://www.encodeproject.org/documents/cede0cbe-d324-4ce7-ace4-f0c3eddf5972/@@download/attachment/ENCODE%20Best%20Practices%20for%20RNA_v2.pdf).
Data exclusions	No data was excluded from our analysis.
Replication	We performed two biological replicates for ATAC-seq and ChIP-seq and three for RNA-seq. All attempts at replication were successful.
Randomization	All the samples were classified based on the genotype, such as WT Col-0 and different PcG mutants.
Blinding	In our study blinding was not relevant as it does not affect the behavior of WT or mutant plants as well as data analysis.

Reporting for specific materials, systems and methods

We require information from authors about some types of materials, experimental systems and methods used in many studies. Here, indicate whether each material, system or method listed is relevant to your study. If you are not sure if a list item applies to your research, read the appropriate section before selecting a response.

Materials & experimental systems

n/a	Involved in the study	
☐	☒	Antibodies
☒	☐	Eukaryotic cell lines
☒	☐	Palaeontology and archaeology
☒	☐	Animals and other organisms
☒	☐	Human research participants
☒	☐	Clinical data
☒	☐	Dual use research of concern

Methods

n/a	Involved in the study	
☐	☒	ChIP-seq
☒	☐	Flow cytometry
☒	☐	MRI-based neuroimaging

Antibodies

Antibodies used	Anti-trimethyl-Histone H3 (Lys27) Antibody, Millipore, Cat: 07-449, Lot: 3146226. Ubiquityl-Histone H2A (Lys119) (D27C4) XP® Rabbit mAb, Cell Signaling Technology, Cat: 8242S, Lot: 6.
Validation	Anti-trimethyl-Histone H3 (Lys27), also known as Anti-H3K27me3, is a highly published Rabbit Polyclonal Antibody. This protein A purified antibody is dot blot tested for trimethylated lysine 27 specificity and validated in WB, ICC, IP.<br>Ubiquityl-Histone H2A (Lys119) (D27C4) XP® Rabbit mAb is validated for:<br>W-Western IP-Immunoprecipitation IHC-Immunohistochemistry ChIP-Chromatin Immunoprecipitation IF-Immunofluorescence F-Flow Cytometry E-P-ELISA-Peptide

ChIP-seq

Data deposition

- [x] Confirm that both raw and final processed data have been deposited in a public database such as GEO.
- [x] Confirm that you have deposited or provided access to graph files (e.g. BED files) for the called peaks.

Data access links
May remain private before publication.

https://www.ncbi.nlm.nih.gov/geo/query/acc.cgi?acc=GSE155378

Files in database submission

ATAC-seq raw data:
atacseq_col0_1_1.fq.gz
atacseq_col0_1_2.fq.gz
atacseq_col0_2_1.fq.gz
atacseq_col0_2_2.fq.gz
atacseq_bmi1abc_1_1.fq.gz
atacseq_bmi1abc_1_2.fq.gz
atacseq_bmi1abc_2_1.fq.gz
atacseq_bmi1abc_2_2.fq.gz
atacseq_clfswn_1_1.fq.gz
atacseq_clfswn_1_2.fq.gz
atacseq_clfswn_2_1.fq.gz
atacseq_clfswn_2_2.fq.gz
atacseq_emf1_1_1.fq.gz
atacseq_emf1_1_2.fq.gz
atacseq_emf1_2_1.fq.gz
atacseq_emf1_2_2.fq.gz
atacseq_lhp1_1_1.fq.gz
atacseq_lhp1_1_2.fq.gz
atacseq_lhp1_2_1.fq.gz
atacseq_lhp1_2_2.fq.gz
atacseq_ring1ab_1_1.fq.gz
atacseq_ring1ab_1_2.fq.gz
atacseq_ring1ab_2_1.fq.gz
atacseq_ring1ab_2_2.fq.gz

ATAC-seq processed data:
atacseq_col0_1.bw
atacseq_col0_2.bw
atacseq_bmi1abc_1.bw
atacseq_bmi1abc_2.bw
atacseq_clfswn_1.bw
atacseq_clfswn_2.bw
atacseq_emf1_1.bw
atacseq_emf1_2.bw
atacseq_lhp1_1.bw
atacseq_lhp1_2.bw
atacseq_ring1ab_1.bw
atacseq_ring1ab_2.bw
consensus_ths.bed

ChIP-seq raw data:
h2aub_col0_1_1.fq.gz
h2aub_col0_1_2.fq.gz
h2aub_col0_2_1.fq.gz
h2aub_col0_2_2.fq.gz

h2aub_emf1_1_1.fq.gz
h2aub_emf1_1_2.fq.gz
h2aub_emf1_2_1.fq.gz
h2aub_emf1_2_2.fq.gz
h2aub_ring1ab_1_1.fq.gz
h2aub_ring1ab_1_2.fq.gz
h2aub_ring1ab_2_1.fq.gz
h2aub_ring1ab_2_2.fq.gz
h3k27me3_col0_1_1.fq.gz
h3k27me3_col0_1_2.fq.gz
h3k27me3_col0_2_1.fq.gz
h3k27me3_col0_2_2.fq.gz
h3k27me3_emf1_1_1.fq.gz
h3k27me3_emf1_1_2.fq.gz
h3k27me3_emf1_2_1.fq.gz
h3k27me3_emf1_2_2.fq.gz
h3k27me3_ring1ab_1_1.fq.gz
h3k27me3_ring1ab_1_2.fq.gz
h3k27me3_ring1ab_2_1.fq.gz
h3k27me3_ring1ab_2_2.fq.gz
h3k27me3_lhp1_1_1.fq.gz
h3k27me3_lhp1_1_2.fq.gz
h3k27me3_lhp1_2_1.fq.gz
h3k27me3_lhp1_2_2.fq.gz
input_col0_1.fq.gz
input_col0_2.fq.gz

ChIP-seq processed data:
h2aub_col0_1.bw
h2aub_col0_2.bw
h2aub_emf1_1.bw
h2aub_emf1_2.bw
h2aub_ring1ab_1.bw
h2aub_ring1ab_2.bw
h3k27me3_col0_1.bw
h3k27me3_col0_2.bw
h3k27me3_emf1_1.bw
h3k27me3_emf1_2.bw
h3k27me3_ring1ab_1.bw
h3k27me3_ring1ab_2.bw
h3k27me3_lhp1_1.bw
h3k27me3_lhp1_2.bw
input_col0.bw
h2aub_consensus_peaks.bed
h3k27me3_consensus_peaks.bed

RNA-seq raw data:
col0_1_1.fq.gz
col0_1_2.fq.gz
col0_2_1.fq.gz
col0_2_2.fq.gz
col0_3_1.fq.gz
col0_3_2.fq.gz
clfswn_1_1.fq.gz
clfswn_1_2.fq.gz
clfswn_2_1.fq.gz
clfswn_2_2.fq.gz
clfswn_3_1.fq.gz
clfswn_3_2.fq.gz
ring1ab_1_1.fq.gz
ring1ab_1_2.fq.gz
ring1ab_2_1.fq.gz
ring1ab_2_2.fq.gz
ring1ab_3_1.fq.gz
ring1ab_3_2.fq.gz
emf1_1_1.fq.gz
emf1_1_2.fq.gz
emf1_2_1.fq.gz

emf1_2_2.fq.gz
emf1_3_1.fq.gz
emf1_3_2.fq.gz

RNA-seq processed data:
gene_expression.tsv

Genome browser session (e.g. UCSC)

http://genome.ucsc.edu/s/fran_rom_cam/Polycomb%20Accesibility

Methodology

Replicates

For all ChIP-seq and ATAC-seq, we did two biological replicates; for RNA-seq, we did three biological replicates.

Sequencing depth

ATAC-seq:

Col0 rep1	44,247,510	18.78%	paired-end	150nt
Col0 rep2	47,788,632	18.80%	paired-end	150nt
bmi1abc rep1	48,364,697	26.32%	paired-end	150nt
bmi1abc rep2	39,317,062	26.03%	paired-end	150nt
clfswn rep1	52,074,011	36.06%	paired-end	150nt
clfswn rep2	32,689,404	28.35%	paired-end	150nt
emf1 rep1	42,209,845	41.99%	paired-end	150nt
emf1 rep2	39,814,391	40.43%	paired-end	150nt
lhp1 rep1	46,171,107	18.71%	paired-end	150nt
lhp1 rep2	48,995,300	19.12%	paired-end	150nt
ring1ab rep1	53,087,907	23.51%	paired-end	150nt
ring1ab rep2	49,457,730	24.74%	paired-end	150nt

ChIP-seq:

Col0 H2Aub rep1	12,780,243	87.24%	paired-end	150nt
Col0 H2Aub rep2	12,641,870	89.26%	paired-end	150nt
emf1 H2Aub rep1	11,204,529	90.54%	paired-end	150nt
emf1 H2Aub rep2	13,164,450	90.90%	paired-end	150nt
ring1ab H2Aub rep1	11,107,370	88.82%	paired-end	150nt
ring1ab H2Aub rep2	11,948,572	86.16%	paired-end	150nt
Col0 H3K27me3 rep1	20,207,084	74.83%	paired-end	150nt
Col0 H3K27me3 rep2	18,237,177	74.82%	paired-end	150nt
emf1 H3K27me3 rep1	21,269,223	73.33%	paired-end	150nt
emf1 H3K27me3 rep2	16,751,109	66.67%	paired-end	150nt
ring1ab H3K27me3 rep1	18,151,696	79.75%	paired-end	150nt
ring1ab H3K27me3 rep2	19,540,914	80.21%	paired-end	150nt
lhp1 H3K27me3 rep1	21,457,122	59.57%	paired-end	150nt
lhp1 H3K27me3 rep2	21,752,391	59.85%	paired-end	150nt

RNA-seq:

Col0 rep1	20,226,425	98.30%	paired-end	150nt
Col0 rep2	21,042,087	98.29%	paired-end	150nt
bmi1abc rep1	54,627,448	97.01%	paired-end	150nt
bmi1abc rep2	46,742,567	96.76%	paired-end	150nt
clfswn rep1	14,890,456	96.60%	paired-end	150nt
clfswn rep2	15,636,978	97.32%	paired-end	150nt
emf1 rep1	20,527,835	98.03%	paired-end	150nt
emf1 rep2	19,557,342	98.54%	paired-end	150nt
ring1ab rep1	19,518,700	98.50%	paired-end	150nt
ring1ab rep2	22,409,408	98.47%	paired-end	150nt

Antibodies

Anti-trimethyl-Histone H3 (Lys27) Antibody, Millipore, Cat: 07-449, Lot: 3146226. Ubiquityl-Histone H2A (Lys119) (D27C4) XP® Rabbit mAb, Cell Signaling Technology, Cat: 8242S, Lot: 6.

Peak calling parameters

```
bowtie2 -p 5 -x $INDEX -1 $SAMPLE_LEFT -2 $SAMPLE_RIGHT -S ${SAMPLE_NAME}.sam
macs2 callpeak -t ${SAMPLE_NAME}.bam -c ${SAMPLE_INPUT} -f BAM --outdir . -n ${_NAME}
```

Data quality

16310 consensus H2Aub peaks
4367 consensus H3K27me3 peaks
23226 consensus THS
Peaks were determined using default parameters independently for each replicate. Only peaks present in both replicates were considered.

Software | MACS2
