# Supplementary material for: H2AK121ub in Arabidopsis associates with a less accessible chromatin state at transcriptional regulation hotspots
Source: Nat Commun. 2021 Jan 12;12:315. doi: 10.1038/s41467-020-20614-1 (PMC7804394; doi:10.1038/s41467-020-20614-1)
Supplement: Supplementary file 4 — Description of Additional Supplementary Files [file 41467_2020_20614_MOESM4_ESM.pdf]

Description of Additional Supplementary Files

Supplementary Data 1.
Localization of THSs in the different genotypes.

Supplementary Data 2.
List of Transcription factors (TFs) used to localize their binding sites. Data accession numbers and references are included.

Supplementary Data 3.
Gene groups in each genotype used for accessibility profiles.

Supplementary Data 4.
Blacklisted regions.
