# Supplementary material for: H2AK121ub in Arabidopsis associates with a less accessible chromatin state at transcriptional regulation hotspots
Source: Nat Commun. 2021 Jan 12;12:315. doi: 10.1038/s41467-020-20614-1 (PMC7804394; doi:10.1038/s41467-020-20614-1)
Supplement: Supplementary file 2 — Peer Review File [file 41467_2020_20614_MOESM2_ESM.pdf]

Reviewer #1 (Remarks to the Author):

The manuscript by Yin et al. analyses the relationship between the activity of PcG proteins in depositing respective histone PTMs and chromatin accessibility at loci subject to these modifications. It targets an important and yet unanswered question in the field of how PcG-associated histone PTMs affect chromatin structure, what is the relationship between the chromatin structure and expression level of the underlying genes and what is the relative contribution of PRC1 and PRC2 activities. The authors analyse two histone PTMs and their combinations – H2AK121ub and H3K27me3, deposited respectively by the PRC1 and PRC2 - in WT and respective mutant plants. Performing ATAC-seq and combining with ChIP-seq and RNA-seq data, they evaluate the state of chromatin accessibility in relation to the presence of the modifications and underlying gene expression. They conclude that H2AK121ub marks less accessible but transcriptionally-permissive chromatin, while H3K27me3 enforces a repressed transcriptionally less-permissive state.

The manuscript addresses a very important question in the field and integrates very valuable datasets, shedding light onto the mechanisms of PcG-mediated chromatin repression. The manuscript is clearly written, comprehensible and methods are well-described allowing reproduction. Still, several analysis approaches and their interpretations are not clear and in my opinion require clarification, discussion and perhaps implementing an alternative approach. These concerns are specified in the points below:

1. It is unclear from the Results or Methods, which profiling data come from the same experiment and which originate from other/previous experiments or publications (see particular examples below). Are the ATAC-seq, ChIP-seq and RNA-seq data all generated using replicates/aliquots of the same biological samples? If not, how compatible are the datasets? In particular:
- 1.1. Pg. 4-5+Fig 1/S2: It is unclear whether the ChIP-seq data used for separating the H2Aub-only/H3K27me3-only and H2Aub-only+H3K27me3 come from WT plants and previous work of the group or from this work – this information does not seem to be given.
- 1.2. Pg.5-6 and Fig 2c-f: It is unclear why the left and right metagene plot in each panel of Fig 2c-f displays different y-axis scale (and even WT differs) – is this data coming from different studies? This information seems to be missing in the results or the methods part.

2. Several analyses demonstrated in Figs 1 and 2 relate to the use of the consensus THS list, which according to the text on Pg. 4 and Methods section are those identified in at least one genotype (Suppl dataset 1: total 17342 THSs, of those 9250 in WT). My concern in general relates to the approach in comparing these to to WT ChIP-seq data (to separate PcG and non-PCG targets) and to what extent the conclusions may be affected by including THSs present ONLY in PcG mutants and modified by H2Aub and/or H3K27me3 in WT, i.e. potentially arising as a consequence of the ABSENCE of these modifications and spurious transcription in the different mutant genotypes:

- 2.1. Pg. 4-5+Fig 1b,d/Fig S2: Consensus THSs are used to determine overall higher accessibility of genes in some of the mutants analysed (depicted by metagene plots of CPM values centred over TSS or THS sites). It this affected by different contribution of the mutant genotypes to the overall number of consensus THSs? Would using only the subset of THSs identified in WT yield the same results?
- 2.2. Pg. 5, Fig 2a: "around 80% of consensus THSs showed an H2AK121ub peak in its vicinity…These results suggest that H2AK121ub1 hallmarks hotspots for transcriptional regulation": Does this statement relate to WT? To what extent may this conclusion be influenced by THSs present only in mutants/at loci with reduced H2AK121ub? This would in contrast indicate that the LOSS/REDUCTION of H2AK121ub results in an emergence of THS? To conclude on whether this is the case in WT, I think individual genotype THSs should also by analysed separately.

3. Pg.5-6 and Fig 2c-f: I am not convinced that the bar charts at the right of each panel fully support the conclusions made:
- 3.1. Pg. 6: bmi1abc is said not to be affected by loss of H3K27me3 on H3K27me3-only targets (Pg5). While this fits the metagene plot on the left of Fig 2d, by looking at the distribution plot on the

right of Fig 2d, a subset of genes seems to be affected (ca 20% genes are at 60% WT level at the most). Is in not in fact comparable situation in ring1ab and H3K27me3 at H3K27me3/H2Aub targets (Pg.5, Fig 2c)? Judging Figs 2c-d, would the conclusion that ring1ab does not seem affected in either 2c or 2d, while bmi1abc is affected in 2c but not 2d be more appropriate? How strong is the support for the general conclusion that "all these proteins (i.e. including RING1A/B and BMI1A/B/C) are required for appropriate H3K27me3 deposition at H3K27me3 AND H2Aub genes (Pg. 5/6) but not at H3K27me3-only genes (Pg6)?
- 3.2. Although the increase of H3K27me3 in emf1-2 is visible in fig2c and d (right charts), Fig S5 shows a very mild-to-moderate increase – how representative is this example? Can increase in H3K27me3 be robustly concluded with respect to the technical limitations of ChIP when it comes to quantitative comparison of two samples, especially without performing a spike-in?

4. Pg.6, par 2: The suggestion that apparent increase in H3K27me3 in emf1-2 may be connected to the different requirement of EMF1 in the root and the shoot and/or relative over-representation of root tissue is speculative. As the expressivity of the "embryonic" phenotype in these mutants can be variable (e.g. Bratzel et al 2010), the penetrance of pickle root (usually itself a not fully penetrant phenotype – e.g. Chen et al 2010, Ogas et al, Aichinger et al….) should be quantified in the individual genotypes by lipid staining to conclude on the relative efficiency of WT-like root formation in emf1-2. In addition, the GO analysis (Fig S5b) may reflect different expression of the photosynthesis-related genes also in the emf1-2 shoot, which from Fig S1 seems less photosynthetically active.

5. Pg.7, par 2 and Supplementary Fig 6: The figure does not show that "a high percentage of H2AK121ub-only genes are transcriptionally active in WT" - the fig shows that in comparison to H2AKub/H3K27me3 or H3K27me3-only genes, these genes are on average more highly transcribed. A comparison with FPKM values of non-PcG genes/active genes is missing, making it impossible to judge on the relative gene activity. In addition, Fig 3a shows no correlation between gene expression and accessibility in the respective H2AKub-depleted categories (left panel), and therefore in my opinion the conclusion of a "role of H2AK121ub in favouring a PERMISSIVE but less accessible chromatin" is disputable.

6. Pg8/9, Fig 4: In the present form, I am not convinced that the data shown in Fig 4 fully supports the conclusion that "increased chromatin accessibility in PcG mutants is not caused by gene expression". As the authors point out, PCA plots in Fig 1 and Fig 4 resemble each other suggesting a relationship between accessibility and gene expression. I think more thorough analysis and interpretation of this data is needed.
- 6.1. Since expression level in connection to increased accessibility is evaluated, a correlation between accessibility and expression level (rather than accessibility and H3K27me3) would seem more logical, either as a correlation plot or by depicting mean expression values of genes in different modification-depletion categories. In Fig 4 in its current form, expression values are reduced to discrete categories (log2 FC +/-1 for altered genes) but trend cannot be evaluated, which may result in misinterpretation.
- 6.2. It is further unclear why the analysis is only done for genes reduced in H3K27me3 (where the accessibility increase with decreasing modification level is less prominent – Fig 3) but not for the H2Aub-reduced genes.
- 6.3. Almost comparable percentage of genes are "upregulated" and "not altered" in bmi1abc and emf1-2, the two mutant genotypes with increased accessibility with reduced H3K27me3. This would imply that ½ of the genes are affected in accessibility AND expression – does this allow the general conclusion quoted above (point 6.)?
- 6.4. In respect to the reasoning on pg 8/9 (considering only genes with less than 60% WT-level of H3K27me3 as they show higher accessibility), what is the reason for including graphs in the main figure for clf swn, lhp1 or ring1ab where accessibility is unchanged?

7. Model Fig 4c: The chromatin state modified by H2Aub is termed "less accessible". While Fig 3 shows that H2Aub PROMOTES lower accessibility, lower accessibility compared to other (non-H2Aub/non-PcG

targets) is not available to allow calling H2Aub-marked chromatin as less-accessible. This could be supported by direct comparison between THS CPM in WT at non-PcG targets, H2Aub-only-targets, H2Aub/H3K27me3 and H3K27me3-only targets.

8. Abstract (pg 2) and Pg 9 – concluding paragraph: It is said that "H2AK121ub marking FAVOURS less accessible chromatin" – in my opinion, the use of the verb "favours" suggests an active choice of the mark (rather than the enzymatic complex) of its deposition site and also that less accessible chromatin is "favoured" (i.e. recruiting?) the modification. I think verbs such as associates with, corresponds to or similar would be more appropriate.

9. Abstract (pg 2): last sentence: …."indicating that gene expression is not always predictive of accessible chromatin"…should state "indicating that accessible chromatin is not always predictive of gene expression"?

10. Abstract (pg.2): The formulation "H2AK121ub and H3K27me3 establish an inaccessible but responsive chromatin TO MARKS LEVELS," is unclear

11. Pg.3: "Several evidence" should spell "Several pieces of evidence"

12. Pg.6 – second paragraph – typo "WT levels= than in lhp1"

13. In general, the resolution of the figures seems low both electronic or printed. This may be due to the pdf conversion but should be improved in the final version of the ms.

Iva Mozgova

Reviewer #2 (Remarks to the Author):

Polycomb group proteins mediate histone modifications and gene repression, but their effect on chromatin structure is not well understood in plants. This manuscript explored chromatin accessibility in mutants deficient for PcG function by performing ATAC-seq. The authors report that THSs (Tn5 hypersensitive sites) co-localize with transcription factor binding sites and nearby H2Aub peaks. The authors also found that decreased H2Aub levels in the bmi1abc mutant correlate with increased accessibility. Concurrent H2Aub and H3K27me3 marks establish an inaccessible but responsive chromatin and H3K27me3 alone form less responsive chromatin. Finally, the authors revealed that chromatin accessibility is not a consequence of changed transcription.

This manuscript reports interesting data on chromatin accessibility in PcG mutants, trying to establish links between histone modifications and the resulting changes in chromatin accessibility. I have three major concerns with this manuscript that will be further detailed below: (i) lack of proper statistical treatment for many of the presented data; (ii) lack of reference to previously published work reaching partly similar conclusions regarding the role of H2Aub in establishing permissive chromatin (Kralemann et al., 2020); and (iii) I fail to see how the authors can distinguish between direct effects of H2Aub on chromatin accessibility versus an effect mediated by BMI1. In Drosophila PRC1 was shown to compact chromatin independently of H2Aub (Francis et al., 2004); therefore, whether the increased accessibility in the bmi1abc mutant is due to BMI1 activity or reduction of H2Aub, remains open and would need to be carefully discussed.

Major comments:
1. Figure 2c-f: the data need to statistically assessed (e.g. presenting them as boxplots and use Mann–Whitney U test to test for differences).

2. Figure 3: Described differences in the text related to this figure need to be statistically tested.
3. Page 4: The authors write that the first identified THSs present in both replicates and then restrict this list to those sites having a CPM larger 3 $q<0.05$ in one of the replicates. Is there any reasoning behind using this threshold?
4. Page 6: "Hence, the apparently increased H3K27me3 levels at these genes may be due to an overrepresentation of the root tissue over other tissues in emf1-2 samples compared to WT." The basis for the argumentation remains unclear to this reviewer.
5. Page 9: "This supports that maintenance of accessible chromatin requires the binding of transcription factors…" I am not convinced that this conclusion is justified; just the fact that that there are TF binding sites does not mean that TFs are binding to those sites. Since the authors find that accessible regions are not necessarily transcribed, is not in support of this statement.
6. Page 4: "This was also the case when analyzing PcG and non-PcG target genes separately". First, the authors need to define PcG and non-PcG target genes. Second, apparently non-PcG target genes have similarly increased chromatin accessibility like PcG targets, suggesting that the gain in chromatin accessibility is not a consequence of PcG protein deficiency, but rather a secondary effect of the phenotype. This requires an explanation.
7. Page 5: "In contrast, this was not evident for H3K27me3 peaks, as despite 60% of consensus THSs showed an H3K27me3 peak within the next 1kb (Fig. 2a, b)"
Fig.2b shows that 60% of THSs are within the next 2kb.
8. Page5: "Nevertheless, analyzing the levels of H3K27me3 at individual genes in ring1ab, we found around 20% of genes with strongly decreased levels (Fig.2c)
It seems around 30% of genes.
9. Page 7: "However, we found that decreasing levels of H2AK121ub led to a progressive increase in accessibility (Fig.3a)" As discussed above, decreasing levels of H2AK121ub may reflect occupancy deficiency of BMI1. Since the increase in accessibility can be the result of decreased BMI1 occupancy, this statement needs adjustment.
10. Fig 3: Despite that H2Aub levels are (apparently) not changed in emf1-2, the accessibility is increased. This requires to be discussed.
11. Page 8: "On the other hand, we did not find significant accessibility changes in lhp1 and ring1ab at any of the gene subsets, which is consistent with the small impact of these mutations on histone marking". As previous described, LHP1 and RING1 are required for H3K27me3 deposition; therefore, the phrase "the small impact of these mutations on histone marking" is not correct. Thus, although H3K27me3 levels are affected, accessibility did not change in lhp1 mutant (even though this needs to be statistically assessed, as outlined above). This needs to be appropriately mentioned and discussed.
12. Fig. 4b: I would like to see data clearly showing the relationship between THS accessibility, H2AK121ub and gene expression to support the conclusion in the last sentence of the abstract and the claim "H2AK121ub/H3K27me3-mediated inaccessible chromatin is however responsive to histone marks levels due to the presence of H2AK121ub-marked hotspots" (Page 9 2nd paragraph).
13. As also pointed out above, the authors need to discuss their data in the context of what is known. For example, in mouse ESCs, PRC1 affect nucleosome spacing but not accessibility, while PRC2 doesn't influence nucleosome spacing or chromatin accessibility (King et al., 2018).

Minor comments:
14. Page 5: "82.71% of these peaks co-localized with H2AK121ub marks"
This needs to be shown.
15. Page 5: "Interestingly, around 80% of consensus THSs showed an H2AK121ub peak in its vicinity (< 1 kb distance from THS) (Fig. 2a)".
<1 kb distance is seen in Fig.2b.
16. Fig. 4a, WT 7 DAG needs to be defined.
17. Fig. 4b: There are two percentage numbers on each panel (top and bottom), please clarify.
18. Methods: ATAC-seq and data analysis, "Nuclei isolation of ATAC-seq experiments were performed as previously described44"
In reference 44, INTACT and sucrose sedimentation are two ways for nuclei isolation. The author should specify which method is used.

19. ChIP-seq and data analysis, please clarify what is used to normalize the H2Aub and H3K27me3 level.
20. RNA-seq and data analysis (bottom), "Specifically, a log2 fold-change of +-1 was used to determine activated, repressed and unaltered genes."What is the criteria for the p-value?
21. In the abstract: "We found that when acting concurrently, H2AK121ub and H3K27me3 establish an inaccessible but responsive chromatin to marks levels…" needs rephrasing, unclear what the authors mean. In general, the term "marks level" should be rephrased.
22. Page 3: Introduction of PRC2 components, should be pointed out that this refers to sporophytic tissue.
23. Page 3: "and nothing is known about the effect of H2AK121ub in plants" This does not reflect the current stage of knowledge, see e.g. Kralemann et al., 2020.
24. Page 6: "The fact that clf28swn7 displayed increased H2AK121ub levels at a number of H2AK121ub/H3K27me3 and only-H2AK121ub marked genes (Fig. 2 e,f) suggests an indirect consequence, as only-H2AK121ub genes are not targeted by these proteins." The sentence is unclear, needs rephrasing.
25. Page 9: "activities. H2AK121ub/H3K27me3-mediated inaccessible chromatin is however responsive to histone marks levels due to the presence of H2AK121ub-marked hotspots (Fig. 4c), which allows gene reprograming." The sentence is unclear, needs rephrasing.
26. Line numbers would have been helpful and should be added.

Reviewer #3 (Remarks to the Author):

In their manuscript, Yin et al., studied the effect of both of H2AK121ub and H3K27me3 histone modifications on chromatin accessibility. By integrating chromatin accessibility data, histone marks and expression analyses in different Arabidopsis PcG mutants, authors observed that H2AK121ub may favors a less accessible chromatin at transcriptional regulation hotspots. They also proposed that when H3K27me3 is alone, chromatin is less responsive, indicating that H2AK121ub-marked hotspots are required for transcriptional responses. Interestingly, authors observed that despite the loss of H2AK121ub and H3K27me3 leads to increased chromatin accessibility, this is not necessarily accompanied by transcriptional activation and they suggested that this indicates that gene expression is not always predictive of accessible chromatin. Overall this study could be potentially interesting. However, at the current state, there are several weak points, which make the paper too preliminary. I listed them below:
1. My major concern is the fact that authors studied the interaction between H2AK121ub and H3K27me3 with no experimental proof that the same nucleosome can harbour both modifications. One can speculate that those two marks could never be on the same nucleosome and what they observed could be due to the fact that they use a mixture of cells and then the interpretation of their correlative analysis would change. To my view, authors must perform ChIPreChIP experiment to validate at least that on some genes both H2AK121ub and H3K27me3 are present on the same nucleosome and then analyze how this could change in PcG mutant context. I think that their conclusion is right now only based on correlation which makes it not convincing enough.
2. The quality of the figure must be improved. For example in the figure 3 is not clear at all and very difficult to understand. Fig 3 panel A why we see only one line for LHP1 and several for bmi abc ? In the figure 2 panel C, D, e and F the WT curve must be in all graphs.
3. To my point of view authors should put more effort in both introduction and discussion of the paper.

Response to Reviewers

Reviewer 1

We would like to thank Dr Mozgova for her valuable and constructive comments and suggestions, which have helped us to improve the manuscript. New and/or modified sections in the revised version of manuscript are highlighted in yellow. In addition, here we provide a detailed point-by-point reply.

1. It is unclear from the Results or Methods, which profiling data come from the same experiment and which originate from other/previous experiments or publications (see particular examples below). Are the ATAC-seq, ChIP-seq and RNA-seq data all generated using replicates/aliquots of the same biological samples? If not, how compatible are the datasets? In particular:

We are sorry for this missing information. Here we indicate the source of the data:

-H2AK121ub and H3K27me3 ChIP-seq data of bmi1abc, clf28swn7 and WT, and H2AK121ub ChIP-seq data of lhp1 at 7 DAG are from previous experiments (Zhou, Romero-Campero et al., 2017).

-H2AK121ub and H3K27me3 ChIP-seq data of WT, ring1ab, emf1-2 and H3K27me3 data of lhp1 at 10 DAG are from and new experiments (this work).

-RNA-seq data of bmi1abc and WT at 7 DAG are from previous results (Zhou, Romero-Campero et al., 2017).

-RNA-seq data of WT, clf28swn7, emf1-2, lhp1, ring1ab at 10 DAG are from new experiments.

-Samples for ATAC-seq (all generated in this work) and for the new ChIP-seq and RNA-seq experiments were grown in parallel under the same conditions and the different biological replicates were collected at 10 DAG for the different analyses.

This is now specified in the Results and Methods sections.

- 1.1. Pg. 4-5+Fig 1/S2: It is unclear whether the ChIP-seq data used for separating the H2Aub-only/H3K27me3-only and H2Aub-only+H3K27me3 come from WT plants and previous work of the group or from this work – this information does not seem to be given.

In this study, we analyzed ChIP-seq data from two different sets of experiments. In order to ensure faithful comparisons, we have treated them separately and combined only the final results. First, the detection of peak marks was performed for each replicate using MACS2 with a specific input for each data set, one for our previously published data corresponding to 7 DAG and another one for our current data corresponding to 10 DAG. Second, the intersection of the replicate peaks were considered for further analysis. And third, although major differences were not detected between 7DAG and 10DAG data in order to considered both experiments we merged the peaks. Peak intersection and merging was performed using bedtools. Then, we separated the different subsets of genes resulting in similar gene lists than the ones previously published[29]. These details are now included in the methods section.

- 1.2. Pg.5-6 and Fig 2c-f: It is unclear why the left and right metagene plot in each panel of Fig 2c-f displays different y-axis scale (and even WT differs) – is this data coming from different studies? This information seems to be missing in the results or the methods part.

Yes, as we indicated above, the data used to generate the left and right metagene plots in each panel of Fig 2c-f come from different studies (Now new Fig 3a-d). When comparing metagene plots and signal levels for each genotype the specific WT data from the corresponding experiment was used as control, never mixing data from the two experiments. These details are now included in the methods section, as well as in the figure legend (see New Fig.3).

2. Several analyses demonstrated in Figs 1 and 2 relate to the use of the consensus THS list, which according to the text on Pg. 4 and Methods section are those identified in at least one genotype (Suppl dataset 1: total 17342 THSs, of those 9250 in WT). My concern in general relates to the approach in comparing these to WT ChIP-seq data (to separate PcG and non-PCG targets) and to what extent the conclusions may be affected by including THSs present ONLY in PcG mutants and modified by H2Aub and/or H3K27me3 in WT, i.e. potentially arising as a consequence of the ABSENCE of these modifications and spurious transcription in the different mutant genotypes:

- 2.1. Pg. 4-5+Fig 1b,d/Fig S2: Consensus THSs are used to determine overall higher accessibility of genes in some of the mutants analysed (depicted by metagene plots of CPM values centred over TSS or THS sites). It this affected by different contribution of the mutant genotypes to the overall number of consensus THSs? Would using only the subset of THSs identified in WT yield the same results?

As you indicate, in WT the number of THSs is lower than in mutants (especially when compared to the strong mutants), as regions that are inaccessible in WT become accessible in mutants. Thus, it is true that the overall accessibility is affected by different contribution of the mutant genotypes to the overall number of consensus THSs. WT THSs are open regions that in general are also accessible in mutants (see New Fig. 1 and Suppl Fig. 2). However, we also noticed a generalized increase in accessibility in the embryonic mutants (bmi1abc, clf28swn7 and emf1-2) compared to vegetative mutants or WT that we considered important to show. In an attempt to reflect this observation, we generated the accessibility profiles at PcG and non-PcG genes in the different genotypes. However, as you indicate, accessibility at non-PcG genes may be indirectly affected, thus, this would be better appreciated showing accessibility profiles at the THSs present in WT in the different genotypes. We have now included these profiles (see New Fig. 1e) and discussed these results (see Pages 6-7). Thank you for the suggestion.

Although this result might suggest that the gain in chromatin accessibility in severe PcG mutants is not a consequence of PcG protein deficiency but rather a secondary effect of the phenotype, we then clearly show that despite basal accessibility is higher in embryonic mutants, loss of PcG function/marking further increases chromatin accessibility (see new Fig. 4).

- 2.2. Pg. 5, Fig 2a: "around 80% of consensus THSs showed an H2AK121ub peak in its vicinity…These results suggest that H2AK121ub1 hallmarks hotspots for transcriptional regulation": Does this statement relate to WT? To what extent may this conclusion be influenced by THSs present only in mutants/at loci with reduced H2AK121ub? This would in contrast indicate that the LOSS/REDUCTION of H2AK121ub results in an emergence of THS? To conclude on whether this is the case in WT, I think individual genotype THSs should also by analysed separately.

We apologize, as it seems that we did not explain clearly this point. We used consensus THSs for the analysis as indeed, a high number of THSs that are not present in WT arises in mutants. These THSs may arise as a direct or indirect consequence of PcG regulation. Thus, to identify the THSs that potentially arise as a direct consequence of the loss of PcG function (either PRC1 or PRC2), we compared the distribution of consensus THSs to that of H2AK121ub and H3K27me3 marked regions

in WT. We have now included a screenshot showing distribution of individual THSs in the different genotypes, consensus THSs and marked regions in WT (see new Fig. 2a).

We in addition found that consensus THSs (some of them present and other not in WT) co-localize with sites enriched for the binding of a wide diversity of TFs as determined by 100 independent ChIP-seq analyses, indicating that consensus THSs are hotspots for transcriptional regulation. Some of these THSs apparently emerge due to the LOSS/REDUCTION of PcG marks. Furthermore, we found that 80% of consensus THSs have an H2AK121ub peak in its vicinity in WT, thus, we propose that that H2AK121ub hallmarks hotspots for transcriptional regulation. We have now tried to clarify these points in the new version of the manuscript (see Pages 7-8).

**We want to mention that when we analyzed the % of consensus THSs with a histone mark peak in its vicinity, by mistake we used not filtered consensus THSs instead of filtered consensus THSs (see methods page 18). We have now corrected this mistake in new Fig. 2b. We found that while results regarding H2AK121ub did not change significantly, the percentage of THSs showing an H3K27me3 peak within the next 2kb was reduced to 40%, which further supports that H2AK121ub hallmarks hotspots for transcriptional regulation.*

3. Pg.5-6 and Fig 2c-f: I am not convinced that the bar charts at the right of each panel fully support the conclusions made:

- 3.1. Pg. 6: bmi1abc is said not to be affected by loss of H3K27me3 on H3K27me3-only targets (Pg5). While this fits the metagene plot on the left of Fig 2d, by looking at the distribution plot on the right of Fig 2d, a subset of genes seems to be affected (ca 20% genes are at 60% WT level at the most). Is in not in fact comparable situation in ring1ab and H3K27me3 at H3K27me3/H2Aub targets (Pg.5, Fig 2c)? Judging Figs 2c-d, would the conclusion that ring1ab does not seem affected in either 2c or 2d, while bmi1abc is affected in 2c but not 2d be more appropriate? How strong is the support for the general conclusion that "all these proteins (i.e. including RING1A/B and BMI1A/B/C) are required for appropriate H3K27me3 deposition at H3K27me3 AND H2Aub genes (Pg. 5/6) but not at H3K27me3-only genes (Pg6)?

You are right. We have now considered that genes displaying strongly reduced levels are those that display less than 60% of WT levels (that is, within 0-40% and 40-60% categories). Therefore, according to this, ring1ab does not seem affected in either Fig. 2c or Fig. 2d (Now New Fig. 3a or 3b), while bmi1abc is affected in Fig. 2c but not in Fig. 2d (Now New Fig. 3a or 3b). However, we wanted to point out that, according to what has been previously reported (Wang et al., 2016), a subset of genes in ring1ab weak mutant indeed displayed strongly reduced levels of H3K27me3 marks at H2AK121ub/H3K27me3 genes (around 7% of these genes; see supplementary Fig. 5), whereas this was not observed at only-H3K27me3 marked genes. The fact that in the ring1ab weak mutant RING1 activity is reduced but not eliminated can explain why H3K27me3 levels are not significantly affected H2AK121ub/H3K27me3 genes. Thus, this and previous data support that all these proteins (including RING1A/B and BMI1A/B/C) are required for appropriate H3K27me3 deposition at H2AK121ub/H3K27me3 marked genes. We have now clarify this in the new version of the manuscript (see Pages 8-9).

- 3.2. Although the increase of H3K27me3 in emf1-2 is visible in fig2c and d (right charts), Fig S5 shows a very mild-to-moderate increase – how representative is this example? Can increase in H3K27me3 be robustly concluded with respect to the technical limitations of ChIP when it comes to quantitative comparison of two samples, especially without performing a spike-in?

In Fig. S5 we wanted to show genes with increased and decreased levels of H3K27me3 compared to WT in the same screenshot. However, there were genes displaying a higher increase that the ones showed in the figure. We have now included more representative examples in which this can be appreciated, and also that this occurs at specific genes and that this is not a general effect (see New Suppl. Fig. 6).

4. Pg.6, par 2: The suggestion that apparent increase in H3K27me3 in emf1-2 may be connected to the different requirement of EMF1 in the root and the shoot and/or relative over-representation of root tissue is speculative. As the expressivity of the "embryonic" phenotype in these mutants can be variable (e.g. Bratzel et al 2010), the penetrance of pickle root (usually itself a not fully penetrant phenotype – e.g. Chen et al 2010, Ogas et al, Aichinger et al….) should be quantified in the individual genotypes by lipid staining to conclude on the relative efficiency of WT-like root formation in emf1-2. In addition, the GO analysis (Fig S5b) may reflect different expression of the photosynthesis-related genes also in the emf1-2 shoot, which from Fig S1 seems less photosynthetically active.

We have now included in Suppl. Fig. 1 Fat Red staining pictures of bmi1abc, clf28swn7 and emf1-2 in which it can be appreciated that emf1-2 mutant is the only one that does not accumulate triacylglycerol in root and develop a WT-like root. Regarding the aerial part, the three mutants accumulate lipids and are less photosynthetically active than WT (we have now included more representative pictures of mutants, see new Suppl. Fig. 1), but this for instance in bmi1abc does not lead to an increase of H3K27me3 to the levels observed in emf1-2. Therefore, based to the phenotype and the reduced levels of H3K27me3 at some genes but not all genes in emf1-2, we propose that EMF1 may be dispensable for H3K27me3 marking in the root (see also New Discussion section page 15). Since we have used whole seedlings for the analyses, we argue that the increased levels of H3K27me3 at specific genes in emf1-2 compared to WT may be a consequence of a higher proportion of root cells in emf1-2 samples than in WT in which both shoot and root develop (See Suppl. Fig. 1c and d). In support of this, GO analysis of the genes with increased levels of H3K27me3 in emf1-2 (for GO analysis we selected the genes according to their mark levels, not expression levels) showed an enrichment in genes involved in photosynthesis and response to light, which are repressed by PcG in WT root (as they already have H3K27me3 marks in WT). As emf1-2 shows a WT-like root, one would expect that these genes may be similarly marked in WT and emf1-2, but they show increased levels, thus this is most probably due to the different shoot/root cell ratio in mutant and WT seedlings. We have now tried to clarify our argument in the new version of the manuscript (see Pages 9-10).

5. Pg.7, par 2 and Supplementary Fig 6: The figure does not show that "a high percentage of H2AK121ub-only genes are transcriptionally active in WT" - the fig shows that in comparison to H2AKub/H3K27me3 or H3K27me3-only genes, these genes are on average more highly transcribed. A comparison with FPKM values of non-PcG genes/active genes is missing, making it impossible to judge on the relative gene activity.

Thanks for the comment. We have now included the box plot showing expression levels of non-PcG marked active genes. This result shows that expression levels of only-H2AK121ub marked genes are significantly higher that H2AK121ub/H3K27me3 or H3K27me3-only genes but also significantly lower than non-PcG genes/active genes (see New Suppl. Fig. 7).

In addition, Fig 3a shows no correlation between gene expression and accessibility in the respective H2AKub-depleted categories (left panel), and therefore in my opinion the conclusion of a "role of H2AK121ub in favouring a PERMISSIVE but less accessible chromatin" is disputable.

In Fig. 3 we analyzed accessibility vs histone marks levels. The fact that decreasing levels of H2AK121ub led to a progressive increase in accessibility at only-H2AK121ub marked genes supports the idea that H2AK121ub associates with a less accessible chromatin (see also accessibility profiles at different subsets of targets in New Fig. 6). On the other hand, as gene expression has been correlated with accessible chromatin, the fact that a considerable number of only-H2AK121ub are transcriptionally active suggest that H2AK121ub at these genes may be associated with a less accessible but still permissive chromatin. We have now tried to clarify this argument in the manuscript (see Page 11 of the new version).

6. Pg8/9, Fig 4: In the present form, I am not convinced that the data shown in Fig 4 fully supports the conclusion that "increased chromatin accessibility in PcG mutants is not caused by gene expression". As the authors point out, PCA plots in Fig 1 and Fig 4 resemble each other suggesting a relationship between accessibility and gene expression. I think more thorough analysis and interpretation of this data is needed.

Gene expression has been positively correlated with chromatin accessibility (as we also show in supplementary fig. 8 for the WT genotype). In strong PcG mutants, there is a high number of upregulated genes (PcG and non-PcG target genes), which therefore may display increased chromatin accessibility. Consequently, it is not surprising to find that PCA analyses in Fig.1 and Fig. 4 (now Supplementary Fig. 9b) resemble each other, as PCA is a dimension reduction method that reduce the size of data by extracting some important information and disposing the rest as noise. Thus, to elucidate if increased chromatin accessibility at PcG targets in PcG mutants is "always" associated to gene expression, we integrated accessibility, mark levels and expression data. If increased chromatin accessibility were always associated to gene expression, the reduced levels of PcG marks and the increased chromatin accessibility in PcG mutants would be mostly associated to gene upregulation. However, we found a comparable percentage of H2AK121ub/H3K27me3 marked genes displaying "upregulated" and "not altered" expression levels in bmi1abc and emf1-2, even though all of them show strongly reduced H3K27me3 levels and increased accessibility. Together these results indicate that while reduced levels of PcG marks are associate with increased accessibility (please note that most dots representing genes are located in the topleft part of the graph), increased accessibility is not always associated with gene expression.

- 6.1. Since expression level in connection to increased accessibility is evaluated, a correlation between accessibility and expression level (rather than accessibility and H3K27me3) would seem more logical, either as a correlation plot or by depicting mean expression values of genes in different modification-depletion categories. In Fig 4 in its current form, expression values are reduced to discrete categories (log2 FC +/-1 for altered genes) but trend cannot be evaluated, which may result in misinterpretation.

As indicated above, we wanted to investigate whether increased chromatin accessibility is always associated with gene expression, not the other way around. If we use the suggested analyses, either the correlation plot or by depicting mean expression values of genes in different modification-depletion, the percentage of genes with unaltered levels of expression will not be appreciated. With our analysis, we can clearly appreciate the percentage of genes with increased, unaltered and decreased levels of expression in relation with marks levels and accessibility. Accordingly, these results revealed that a very high percentage of genes displayed unaltered expression levels but reduced levels of PcG marks and increased accessibility.

- 6.2. It is further unclear why the analysis is only done for genes reduced in H3K27me3 (where the accessibility increase with decreasing modification level is less prominent – Fig 3) but not for the H2Aub-reduced genes.

Thank you for the suggestion. We have now included in the figure (Now new Fig. 5) the integration results obtained at the three subset of PcG marked genes in bmi1abc, clf28swn7 and emf1-2, and removed ring1ab and lhp1 as accessibility does not change in these mutants. These results confirm that despite the loss of H2AK121ub at only-H2AK121ub also affect chromatin accessibility, this not always leads to upregulation of gene expression.

- 6.3. Almost comparable percentage of genes are "upregulated" and "not altered" in bmi1abc and emf1-2, the two mutant genotypes with increased accessibility with reduced H3K27me3. This would imply that ½ of the genes are affected in accessibility AND expression – does this allow the general conclusion quoted above (point 6.)?

This implies that ½ or even a higher proportion of the genes are affected in accessibility but not in expression, supporting that "increased chromatin accessibility in PcG mutants is not caused by gene expression"

- 6.4. In respect to the reasoning on pg 8/9 (considering only genes with less than 60% WT-level of H3K27me3 as they show higher accessibility), what is the reason for including graphs in the main figure for clf swn, lhp1 or ring1ab where accessibility is unchanged?

According to reviewer suggestion, we have removed ring1ab and lhp1 results from the figure (see New Fig. 5).

7. Model Fig 4c: The chromatin state modified by H2Aub is termed "less accessible". While Fig 3 shows that H2Aub PROMOTES lower accessibility, lower accessibility compared to other (non-H2Aub/non-PcG targets) is not available to allow calling H2Aub-marked chromatin as less-accessible. This could be supported by direct comparison between THS CPM in WT at non-PcG targets, H2Aub-only-targets, H2Aub/H3K27me3 and H3K27me3-only targets.

Thanks for the suggestion. We have now included the accessibility profile at TSS of non-PcG targets active genes, only-H2AK121ub targets, H2Aub/H3K27me3 and only-H3K27me3 targets in WT in new Fig. 5 together with the proposed model.

8. Abstract (pg 2) and Pg 9 – concluding paragraph: It is said that "H2AK121ub marking FAVOURS less accessible chromatin" – in my opinion, the use of the verb "favours" suggests an active choice of the mark (rather than the enzymatic complex) of its deposition site and also that less accessible chromatin is "favoured" (i.e. recruiting?) the modification. I think verbs such as associates with, corresponds to or similar would be more appropriate.

We have changed along the manuscript the verb "favour" to "associate" according to your suggestion.

9. Abstract (pg 2): last sentence: …."indicating that gene expression is not always predictive of accessible chromatin"…should state "indicating that accessible chromatin is not always predictive of gene expression"?

Corrected

10. Abstract (pg.2): The formulation "H2AK121ub and H3K27me3 establish an inaccessible but responsive chromatin TO MARKS LEVELS," is unclear

We have removed "to marks levels".

11. Pg.3: "Several evidence" should spell "Several pieces of evidence"

Corrected

12. Pg.6 – second paragraph – typo "WT levels= than in lhp1"

Corrected (paragraph changes).

13. In general, the resolution of the figures seems low both electronic or printed. This may be due to the pdf conversion but should be improved in the final version of the ms.

We apologize for this. We have now provided better quality Figures.

Reviewer 2

We would like to thank the reviewer for his/her detailed comments and suggestions. They were extremely useful to improve the manuscript. In relation to the three major concerns raised by the reviewer, we would like to indicate:

(i) We have now provided proper statistical treatment for the presented data (see response to reviewer comments).

(ii) We apologize for this, but by the time we finished and submitted the manuscript, we did not find the work at NCBI. Although the manuscript was sent to reviewers on July 21, we submitted the manuscript much earlier. First, it was submitted to Nature Genetics and then it was transferred to Nat Comm, which took some time. In addition, the initial evaluation of the manuscript by Nat Comm took longer than usual due to the situation caused by the pandemic. We have now referenced the work and discussed it in relation to our results (see introduction and discussion sections).

(iii) While it is true that we cannot distinguish between direct effects of H2AK121ub on chromatin accessibility versus an effect mediated by BMI1 with our approach, some pieces of evidence suggest that H2AK121ub is required for this effect (see response to comment 9 and new discussion section). Regarding Drosophila PRC1 chromatin compaction ability, it has been shown that this is mediated by the c-terminal region of Psc, which display functional homology to EMF1 (Calonje et al., 2008; Beh et al., 2011). Although Psc combines in one polypeptide BMI1 and EMF1 functions, it is the c-terminal region the one involved in chromatin compaction. In vertebrates, Psc/EMF1 chromatin compaction ability has been shown to be carried out by Cbx2 (M33) (Grau et al. 2011), which is a Pc homolog and not a BMI1 one. Accordingly, besides H2AK121ub, we found that EMF1 also plays a role in regulating chromatin accessibility. Interestingly, while in animals Psc/EMF1/Cbx2-ability or function is linked to PRC1, in plants it turned out to be associated with PRC2, as EMF1 co-purify with this complex. We have discussed these points in the new discussion section.

Bellow you will find the point-by-point response to reviewer comments. New and/or modified sections in the revised version of manuscript are highlighted in yellow.

Major comments:

1. Figure 2c-f: the data need to statistically assessed (e.g. presenting them as boxplots and use Mann–Whitney U test to test for differences).

We have now included boxplots and performed Mann-Whitney-Wilcox non parametric test to assess significance (see new Fig. 3).

2. Figure 3: Described differences in the text related to this figure need to be statistically tested.

We have performed and Included the assessment for significance (see new Fig. 4).

3. Page 4: The authors write that the first identified THSs present in both replicates and then restrict this list to those sites having a CPM larger 3 q<0.05 in one of the replicates. Is there any reasoning behind using this threshold?

We estimated the level of background signal in our ATAC-seq data by randomly generating regions outside THS, finding that some THS although fullfilled the statistical threshold q < 0.05 presented a signal close to the background level. In ATAC-seq data analysis, we lack an equivalent for input sample as we have for ChIP-seq making difficult to distinguish between signal and background noise. In order to attenuate this, we computed the 5% percentile of the accessibility signal in THS in all genotypes, which was approximately 3 CPM in all of them, and discarded the extreme values below this threshold. This has been now included in the Methods section.

4. Page 6: "Hence, the apparently increased H3K27me3 levels at these genes may be due to an overrepresentation of the root tissue over other tissues in emf1-2 samples compared to WT." The basis for the argumentation remains unclear to this reviewer.

We have now included in Suppl. Fig. 1 Fat Red staining pictures of bmi1abc, clf28swn7 and emf1-2 in which it can be appreciated that emf1-2 mutant is the only one that does not accumulate triacylglycerol in root and develop a WT-like root. Conversely, the aerial part of the three mutants display stunted development and accumulate lipids (see new Suppl. Fig. 1). Therefore, based to the phenotype and the reduced levels of H3K27me3 at some genes but not all genes in this mutant, we propose that EMF1 may be dispensable for H3K27me3 marking in the root (see also New Discussion section page 15). Since we have used whole seedlings for the analyses, we argue that the increased levels of H3K27me3 at specific genes in emf1-2 compared to WT may be a consequence of a higher proportion of root cells in emf1-2 samples than in WT, in which both shoot and root develop (See Suppl. Fig. 1c and d). In support of this, GO analysis of the genes with increased levels of H3K27me3 in emf1-2 (for GO analysis we selected the genes according to their mark levels, not expression levels) showed an enrichment in genes involved in photosynthesis and response to light, which are repressed by PcG in WT root as they have H3K27me3 marks. As emf1-2 shows a WT-like root, one would expect that these genes may be similarly marked in WT and emf1-2, but they show increased levels, thus we argue that this is most probably due to the different shoot/root cell ratio in mutant and WT samples. We have now tried to clarify our argument in the new version of the manuscript (see Pages 9-10).

5. Page 9: "This supports that maintenance of accessible chromatin requires the binding of transcription factors…" I am not convinced that this conclusion is justified; just the fact that that

there are TF binding sites does not mean that TFs are binding to those sites. Since the authors find that accessible regions are not necessarily transcribed, is not in support of this statement.

The reviewer is right. We have corrected the paragraph.

In any case, please note that the TF binding sites has been determined in this study by reanalyzing in a uniform manner 100 independent ChIP-seq data sets previously published covering most TF families in Arabidopsis thaliana. In supplementary Dataset 2 we list the details for each ChIP-seq data set, providing evidence of the binding of the corresponding TF to specific regions in the genome.

6. Page 4: "This was also the case when analyzing PcG and non-PcG target genes separately". First, the authors need to define PcG and non-PcG target genes. Second, apparently non-PcG target genes have similarly increased chromatin accessibility like PcG targets, suggesting that the gain in chromatin accessibility is not a consequence of PcG protein deficiency, but rather a secondary effect of the phenotype. This requires an explanation.

PcG genes are those genes associated with H2AK121ub and/or H3K27me3 peaks in WT, and non-PcG target genes are those lacking PcG marks.

Since we noticed a phenotype-effect on chromatin accessibility in embryonic mutants, we wanted to take this into consideration in order to evaluate if loss of PcG function actually leads to changes in chromatin accessibility. To reflect this effect, we have now used a better way: Since accessibility at non-PcG genes may be indirectly affected by activation of PcG targets, we now generated accessibility profiles at the THSs of WT in the different genotypes (see New Fig. 1e). These profiles show that open regions in WT get even more accessible in embryonic mutants (see Pages 6-7). Nevertheless, although this result, as reviewer indicates, a priori might suggest that the gain in chromatin accessibility in these mutants is only a secondary effect of the phenotype, we then clearly show that loss of PcG function/marking indeed leads to further increased accessibility (see new Fig. 4).

7. Page 5: "In contrast, this was not evident for H3K27me3 peaks, as despite 60% of consensus THSs showed an H3K27me3 peak within the next 1kb (Fig. 2a, b)"

Fig.2b shows that 60% of THSs are within the next 2kb.

Thanks for the comment. We have corrected this mistake. In addition, we realized that for this analysis we used by mistake "not filtered" consensus THSs instead of "filtered" consensus THSs (see methods page 18). We have now corrected this mistake in new Fig. 2b. We found that while results regarding H2AK121ub did not significantly change, the percentage of THSs showing an H3K27me3 peak within the next 2kb was reduced to 40%, which further supports that H2AK121ub hallmarks hotspots for transcriptional regulation.

8. Page5: "Nevertheless, analyzing the levels of H3K27me3 at individual genes in ring1ab, we found around 20% of genes with strongly decreased levels (Fig.2c)

30%....

We have changed the description of Fig. 2c-f results (New Fig. 3a-d. See Page 8-10 of the new version). We have now considered that genes displaying strongly reduced levels are those that display less than 60% of WT levels (that is, within 0-40% and 40-60% categories). Therefore, according to this, around 7% of H2AK121ub/H3K27me3 genes show strongly affected levels of H3K27me3 levels in ring1ab (Fig. 3a).

9. Page 7: "However, we found that decreasing levels of H2AK121ub led to a progressive increase in accessibility (Fig.3a)" As discussed above, decreasing levels of H2AK121ub may reflect occupancy deficiency of BMI1. Since the increase in accessibility can be the result of decreased BMI1 occupancy, this statement needs adjustment.

It is true that we cannot discern whether the effect is caused by decreased levels of H2AK121ub marks or by occupancy deficiency of BMI1. However, the fact that in clf28swn7, in which BMI1 is presumably present at only-H2AK121ub genes as in WT, we see an effect of the levels of H2AK121ub at only-H2AK121ub genes (higher levels==lower accessibility), suggests that H2AK121ub is playing a direct role. In any case, we have contemplated this possibility in the discussion of the revised version.

10. Fig 3: Despite that H2Aub levels are (apparently) not changed in emf1-2, the accessibility is increased. This requires to be discussed.

We have discussed this result. See Discussion section, Page 15-16.

11. Page 8: "On the other hand, we did not find significant accessibility changes in lhp1 and ring1ab at any of the gene subsets, which is consistent with the small impact of these mutations on histone marking". As previous described, LHP1 and RING1 are required for H3K27me3 deposition; therefore, the phrase "the small impact of these mutations on histone marking" is not correct. Thus, although H3K27me3 levels are affected, accessibility did not change in lhp1 mutant (even though this needs to be statistically assessed, as outlined above). This needs to be appropriately mentioned and discussed.

We have now corrected and clarified this point. See results (Page 9 and 12). In addition, we have included statistical assessment for significance of these results (see New Fig.3 and 4).

12. Fig. 4b: I would like to see data clearly showing the relationship between THS accessibility, H2AK121ub and gene expression to support the conclusion in the last sentence of the abstract and the claim "H2AK121ub/H3K27me3-mediated inaccessible chromatin is however responsive to histone marks levels due to the presence of H2AK121ub-marked hotspots" (Page 9 2nd paragraph).

We have now included graphs showing the integration results of accessibility, H2AK121ub and gene expression in bmi1abc, clf28swn7 and emf1-2 to support this claim. Furthermore, we also included the ones considering only-H3K27me3, which further support that the presence of H2AK121ub-marked hotspots are required for transcriptional responses (see New Fig. 5).

13. As also pointed out above, the authors need to discuss their data in the context of what is known. For example, in mouse ESCs, PRC1 affect nucleosome spacing but not accessibility, while PRC2 doesn't influence nucleosome spacing or chromatin accessibility (King et al., 2018).

We have now discussed our results in relation with this work and others (See new Discussion section).

Minor comments:

14. Page 5: "82.71% of these peaks co-localized with H2AK121ub marks" show it

We have now included a Venn diagram to show this (Fig. 2c). However, as mentioned in comment 7, we now used for the analysis filtered-consensus THSs, finding that 96% of these regions co-localized with H2AK121ub.

15. Page 5: "Interestingly, around 80% of consensus THSs showed an H2AK121ub peak in its vicinity (< 1 kb distance from THS) (Fig. 2a)".

<1 kb distance is seen in Fig.2b.

Corrected

16. Fig. 4a, WT 7 DAG needs to be defined.

Although this figure has been moved to supplementary information (Suppl. Fig. 9b), we have now defined this in the figure and figure legend. In addition, we have indicated in the methods section that RNA-seq data of bmi1abc and WT at 7 DAG have been previously published[29] and that RNA-seq data of WT and the remaining mutants at 10 DAG were generated for this work.

17. Fig. 4b: There are two percentage numbers on each panel (top and bottom), please clarify.

Top percentage refer to the genes with up-, not altered or down-regulated expression that loss more than 60% of WT levels and display increased accessibility, while bottom percentage indicate genes with up-, not altered or down-regulated expression that loss more than 60% of WT levels and display reduced accessibility. (please note that most dots representing genes are located in the topleft part of the graph). This is now indicated in the figure legend (see New Fig. 5 legend).

18. Methods: ATAC-seq and data analysis, "Nuclei isolation of ATAC-seq experiments were performed as previously described44" In reference 44, INTACT and sucrose sedimentation are two ways for nuclei isolation. The author should specify which method is used.

We used the sucrose sedimentation protocol. It is now indicated in Methods section.

19. ChIP-seq and data analysis, please clarify what is used to normalize the H2Aub and H3K27me3 level.

In this study, we analyzed ChIP-seq data from two different experiments. In order to ensure faithful comparisons and avoid major issues related to normalization we have treated them separately and combined only the final results. When comparing metagene plots and signal levels for each genotype the specific WT data from the corresponding experiment was used as control, never mixing data from the two experiments. Specifically, the function bamCoverage was used to compute normalized signal levels using the parameter –normalizeUsing CPM. These details are now included in the methods section.

20. RNA-seq and data analysis (bottom), "Specifically, a log2 fold-change of +-1 was used to determine activated, repressed and unaltered genes."What is the criteria for the p-value?

A log2 fold-change of ± 1 and a p-value of 0.05 was used. This is now indicated in the methods section.

21. In the abstract: "We found that when acting concurrently, H2AK121ub and H3K27me3 establish an inaccessible but responsive chromatin to marks levels…" needs rephrasing, unclear what the authors mean. In general, the term "marks level" should be rephrased.

This has been corrected along the manuscript.

22. Page 3: Introduction of PRC2 components should be pointed out that this refers to sporophytic tissue.

Corrected

23. Page 3: "and nothing is known about the effect of H2AK121ub in plants" This does not reflect the current stage of knowledge, see e.g. Kralemann et al., 2020.

We wanted to indicate that nothing is known about the effect of H2AK121ub on chromatin accessibility in plants. In any case, we have included Kralemann et al., 2020 reference and discussed our results in relation with their results.

24. Page 6: "The fact that clf28swn7 displayed increased H2AK121ub levels at a number of H2AK121ub/H3K27me3 and only-H2AK121ub marked genes (Fig. 2 e,f) suggests an indirect consequence, as only-H2AK121ub genes are not targeted by these proteins." The sentence is unclear, needs rephrasing.

Changed by: The fact that clf28swn7 displayed increased H2AK121ub levels at a number of H2AK121ub/H3K27me3 and only-H2AK121ub marked genes (Fig. 3c,d) suggests an indirect consequence, since this effect was observed at only-H2AK121ub genes that are not CLF or SWN targets[45] (see Page 10).

25. Page 9: "activities. H2AK121ub/H3K27me3-mediated inaccessible chromatin is however responsive to histone marks levels due to the presence of H2AK121ub-marked hotspots (Fig. 4c), which allows gene reprograming." The sentence is unclear, needs rephrasing.

Changed by: we also propose that chromatin at H2AK121ub/H3K27me3 marked genes associates with inaccessible but responsive chromatin, most likely due to the presence of H2AK121ub-marked transcriptional regulation hotspots, which may be important to allow gene reprogramming (Fig. 6). Conversely, chromatin at only-H3K27me3 marked genes, which is generally not associated with these hotspots, is less responsive (Fig. 6), suggesting that the lack of transcriptional hotspots may prevent the ectopic expression of these genes (see Page 16).

26. Line numbers would have been helpful and should be added.

We apologize for that. We have added line numbers in the new version of the manuscript.

Reviewer 3

Bellow you will find the point-by-point response to reviewer comments. New and/or modified sections in the revised version of manuscript are highlighted in yellow.

1. My major concern is the fact that authors studied the interaction between H2AK121ub and H3K27me3 with no experimental proof that the same nucleosome can harbour both modifications. One can speculate that those two marks could never be on the same nucleosome and what they observed could be due to the fact that they use a mixture of cells and then the interpretation of their correlative analysis would change. To my view, authors must perform ChIPreChIP experiment to validate at least that on some genes both H2AK121ub and H3K27me3 are present on the same nucleosome and then analyze how this could change in PcG mutant context. I think that their conclusion is right now only based on correlation which makes it not convincing enough.

Although it has been extensively reported the combined action of PRC1 and PRC2 in both animals and plants, we have performed ChIPreChIP experiment to validate the presence of H2AK121ub and H3K27me3 marks at some H2AK121ub/H3K27me3 marked genes in comparison to only-H3K27me3 marked genes (see New Supplementary Fig. 4). In any case, we point out in the new version of the manuscript that despite H2AK121ub and H3K27me3 marks often co-localize at genes, H3K27me3

marked peaks are generally much longer than that of H2AK121ub, indicating that nucleosomes marked with both modifications are located at specific regions (see the new introduction section) . We hope that these results address reviewer concerns.

2. The quality of the figure must be improved. For example in the figure 3 is not clear at all and very difficult to understand. Fig 3 panel A why we see only one line for LHP1 and several for bmi abc ? In the figure 2 panel C, D, e and F the WT curve must be in all graphs.

We apologize for this. We have now provided better quality Figures.

Regarding the question: "why we see only one line for lhp1 and several for bmi1abc in Fig. 3 panel A (Fig. 4 in the new version)"?
Fig. 3 (now Fig.4) represents the accessibility profile at the region surrounding the TSS of gene groups divided according to their levels of histone marks relative to WT in mutants. Thus, in the different mutants, each line represents a gene group with a different level of marks. We indicated in the figure legend that gene groups with a very small number of genes were excluded as they produced noisy profiles. In Supplementary Dataset 3, we indicated which groups were used in each case. Therefore, in Fig. 3a (now Fig. 4a) as the number of genes with strongly altered levels of H2AK121ub in lhp1 are very few, we only represented the groups of 60-80%, 80-120% and >120%, where 80-120% group corresponds to WT-like levels. These three groups display overlapping profiles (for this it seems like there is only one line), indicating that their differences in the levels of the marks do not lead to changes in their accessibility profile. Conversely, in bmi1abc, each group is well represented and their accessibility profiles are significantly different. These also applies for other cases in which can be observed few number of profile lines.

Regarding the comment: In the figure 2 panel C, D, e and F the WT curve must be in all graphs:
We apologize but we do not understand exactly what means this comment, as WT curve was in all graphs (see blue lines in the four graphs). The two different panels of metagene plots in section C, D, E and F of Fig. 2 (now New Fig. 3a-d) correspond to independent sets of experiments, each one with its WT control. This has been clarified in the new version of the manuscript (see Page 8 and Methods section)

3. To my point of view authors should put more effort in both introduction and discussion of the paper.

According to reviewer suggestion, we have now included more elaborated introduction and discussion sections.

Reviewer #1 (Remarks to the Author):

This is a second submission of the manuscript by Xiaochang Yin, Francisco J Romero-Campero et al. In the revised version of the manuscript, the authors have in my opinion carefully considered and addressed all the reviewers´ comments and modified the manuscript accordingly. Most figures have been modified to accommodate new supporting results panels and statistical analyses and the text has been accordingly changed to further increase the clarity of the manuscript.
My comments and enquiries have been fully addressed in this manuscript version and where necessary, the manuscript text/figures have been changed to reflect the discussion - thank you.

Reviewer #2 (Remarks to the Author):

The authors addressed many of my comments and improved the manuscript. There remain however a couple of points to be addressed, in particular related to the new figure 5, which is a central part of this manuscript:
Figure 5 and lines 285ff: The authors want to show a correlation between loss of epigenetic modifications and increased accessibility. In order to do so, they need to make a statistical test for correlation and discuss possible differences in the correlation in relation to the statements they make.
Figure 5, panels a and c: The authors marked genes that were deregulated; but apparently they used a cutoff, as the color code starts for genes with a logFC of changes in epigenetic modifications of -1. If there is a reason to do so, this needs to be explained or done without cutoff.
Line 830: If, as the authors wrote, the numbers refer to the percentage of genes displaying altered or unaltered expression levels, then this should add up to 100%. The authors need to specify precisely what the numbers refer to.
Line 422 and others: Name of test is Mann-Whitney-Wilcoxon test
Line 827: Should be panel c not b
Line 302: The role of H2AK121ub in gene repression was not questioned by Kralemann et al.; in this work, it was shown that PRC1-mediated H2Aub1 is associated with gene responsiveness, and its repressive function requires PRC2 recruitment. This needs to be clarified.
Line 38ff: The word "interestingly" is not really justified, it is not a new discovery that accessible chromatin is not necessarily connected to transcription.
Line 323ff: I do not follow the logic of the argument; based on the data provided, the authors cannot disentangle between a functional role of BMI or H2AK121ub in regulating chromatin accessibility.

Reviewer #3 (Remarks to the Author):

I am happy that the authors have made the changes requested and have no further issues to raise.

Response to Reviewer #2

The authors addressed many of my comments and improved the manuscript. There remain however a couple of points to be addressed, in particular related to the new figure 5, which is a central part of this manuscript:

1- Figure 5 and lines 285ff: The authors want to show a correlation between loss of epigenetic modifications and increased accessibility. In order to do so, they need to make a statistical test for correlation and discuss possible differences in the correlation in relation to the statements they make.

We have now added to the new version of the manuscript the Supplementary Figure 10 in which this information is included. The figure includes Scatter plots showing the relationship between accessibility at THS and levels of H2K121ub or H3K27me3 marks at PcG marked genes in bmi1abc, clf28swn7 and emf1-1. In each case, the correlation coefficient (r) and pvalue according to F test are indicated. Percentage of genes with increased or decreased levels of accessibility and histone marks are also indicated. In addition, box plots showing accessibility and expression changes in genes grouped according to their levels of H2AK121ub or H3K27me3 marks relative to WT (0-40%, 40-60%, 60-80%, 80-120% and >120%) are included. Significant differences between groups are indicated according to Mann-Whitney-Wilcoxon test.

Note that except for the case of only-H2AK121ub marked genes, in all cases there is weak but significant negative correlation between the levels of histone marks and accessibility. Nevertheless, comparison of accessibility changes among the different only-H2AK121ub gene groups in bmi1abc also indicates a negative relationship between H2AK121ub levels and accessibility. The fact that this correlation is not evident in the subset of only-H2AK121ub genes is most probably because the genes that do not lose marks (80-120% and >120% groups) show a higher degree of accessibility than the same groups of genes in the other subsets of PcG marked genes, which reduce the strength of the linear relationship. Furthermore, it is important to take into account that the three embryonic mutants display a "globally increased chromatin accessibility compared to WT seedlings" (as indicated in the manuscript), which may reduce the strength of the linear relationship in all cases, as all genes, including those that do not lose marks, display increased accessibility in mutants compared to WT. Nevertheless, in spite of this we can still appreciate a negative correlation between the levels of histone marks and accessibility.

We hope that this will address the point raised by the reviewer.

2- Figure 5, panels a and c: The authors marked genes that were deregulated; but apparently they used a cutoff, as the color code starts for genes with a logFC of changes in epigenetic modifications of -1. If there is a reason to do so, this needs to be explained or done without cutoff.

Since accessibility changes at only-H2AK121ub, H2AK121ub/H3K27me3 or only-H3K27me3 marked genes in mutants were evident at genes displaying less than 60% of WT H2AK121ub and/or H3K27me3 levels (Figure 4), we analyzed whether or not the expression of the genes with less than 60% of WT marks was affected. Therefore, we

used the cutoff log2FC ≤ -0.74, which correspond to 60% of WT levels. We have now clearly explained this in the new version of the manuscript (lines 281-286 and Figure 5 legend). The cutoff is visible in Figure 5a and c as in bmi1abc and emf1-2 there are genes having more than 60% of WT levels, but not in b, as in clf28swn7 all genes display less than 60% of WT levels.

3- Line 830: If, as the authors wrote, the numbers refer to the percentage of genes displaying altered or unaltered expression levels, then this should add up to 100%. The authors need to specify precisely what the numbers refer to.

We apologize for including some wrong percentages. As reviewer indicates, the sum in each panel should be 100%. We have corrected this. We have also specified in Figure 5 legend that the sum of top and bottom numbers from the same panel represent 100 % of the genes with less than 60% of WT H2K121ub or H3K27me3 and altered accessibility compared to WT. Thanks.

4- Line 422 and others: Name of test is Mann-Whitney-Wilcoxon test

Corrected along the manuscript. Thanks.

5- Line 827: Should be panel c not b

Corrected. Thanks.

6- Line 302: The role of H2AK121ub in gene repression was not questioned by Kralemann et al.; in this work, it was shown that PRC1-mediated H2Aub1 is associated with gene responsiveness, and its repressive function requires PRC2 recruitment. This needs to be clarified.

This has been now clarified. See 301-311 new paragraph.

7- Line 38ff: The word "interestingly" is not really justified, it is not a new discovery that accessible chromatin is not necessarily connected to transcription.

Changed. See new Abstract.

8- Line 323ff: I do not follow the logic of the argument; based on the data provided, the authors cannot disentangle between a functional role of BMI or H2AK121ub in regulating chromatin accessibility.

It is true that we cannot rule out this possibility. We have clearly indicate this in the manuscript. However, we also indicate that the fact that we observed accessibility changes associated with the levels of H2AK121ub in clf28swn7, in which the expression of the BMI1s is not altered, and the recruitment of the BMI1s should not be affected (as PRC1 recruitment is independent of PRC2), suggests the participation of H2AK121ub.

Reviewer #2 (Remarks to the Author):

The authors have addressed my concerns.
